# Supplementary material for: NFATC1 dysfunction-triggered MSC senescence induces tooth aging amenable to senolytic therapy
Source: Stem Cell Reports. 2026 May 21;21(6):102925. doi: 10.1016/j.stemcr.2026.102925 (PMC13261951; doi:10.1016/j.stemcr.2026.102925)
Supplement: Document S1. Figures S1–S5 and Tables S1–S4 [file mmc1.pdf]

Stem Cell Reports, Volume *21*

Supplemental Information

NFATC1 dysfunction-triggered MSC senescence induces tooth aging amenable to senolytic therapy

Feifei Li, Changhao Yu, Lin Yao, Yawen Tang, Xue Yang, Yitian Wang, Jianxin Liu, Bei Yin, Haisheng Wang, and Fanyuan Yu

SUPPLEMENTAL FIGURES

Figure S1. Validation of *NFATC1* knockdown in promoting senescence of hDPC.

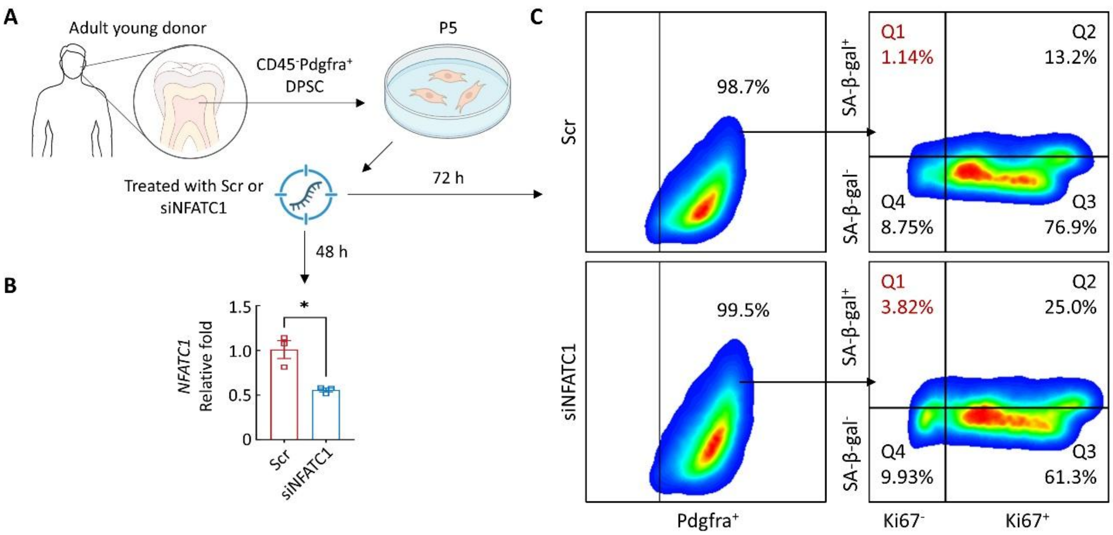

(A) Experimental workflow for siRNA-mediated NFATC1 knockdown in hDPSC. (B) Validation of siNFATC1 knockdown efficiency by RT-qPCR. (C) Flow cytometric analysis of senescent cell fraction following *NFATC1* knockdown.

* $p < 0.05$

Figure S2. Extended data for NFATC1-expressing dental pulp MSCs ablation models.

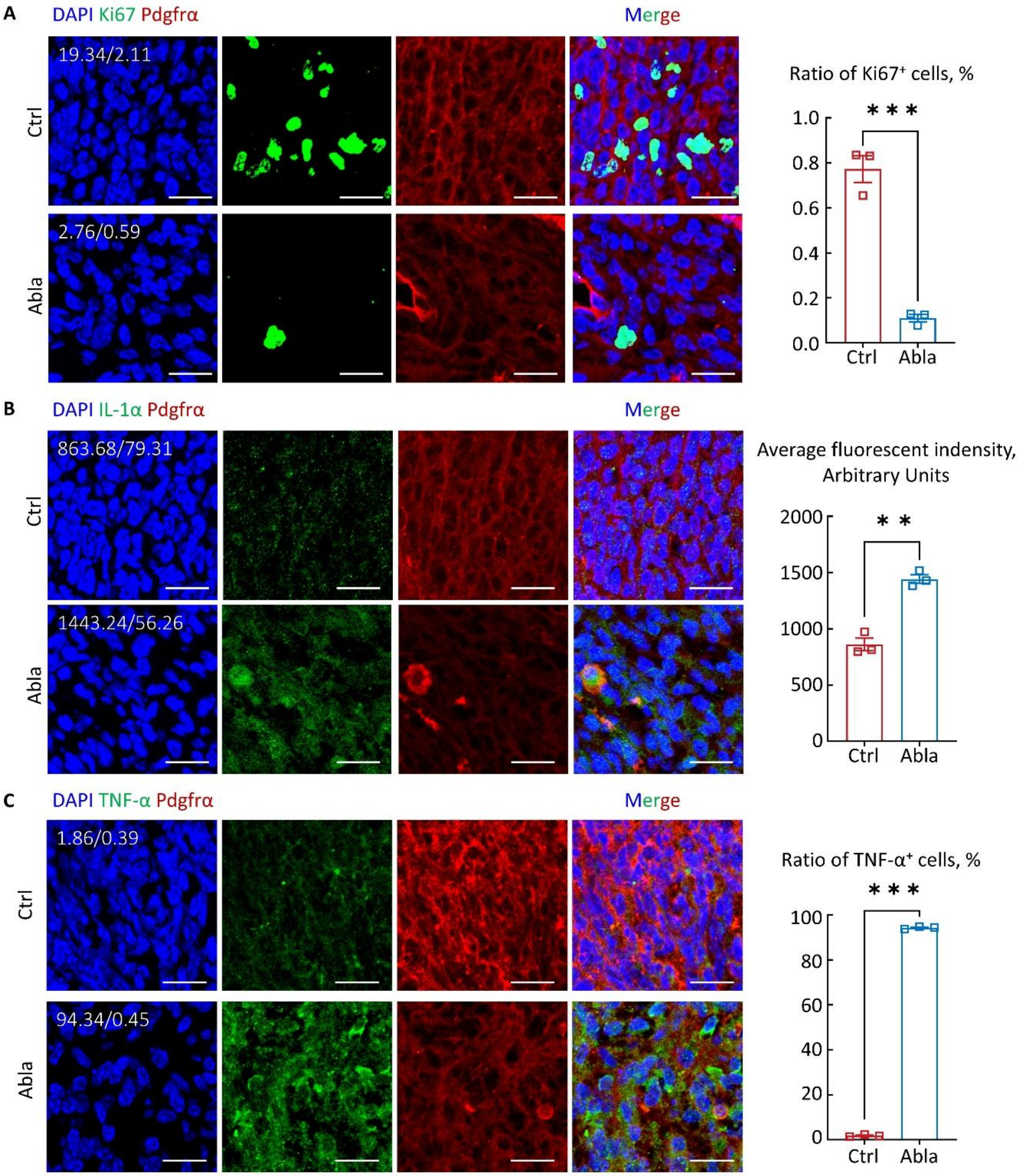

(A-C) Representative IF images of tracing and ablation, detecting the levels of Ki67, TNF-α, and Il-1α. The statistical data of IF were provided in each panel with mean/SEM. Scale bars, 200 μm. n = 3 per group.

** $p < 0.01$, *** $p < 0.005$

Figure S3. Extended data for the gene manipulation combined with tracing model.

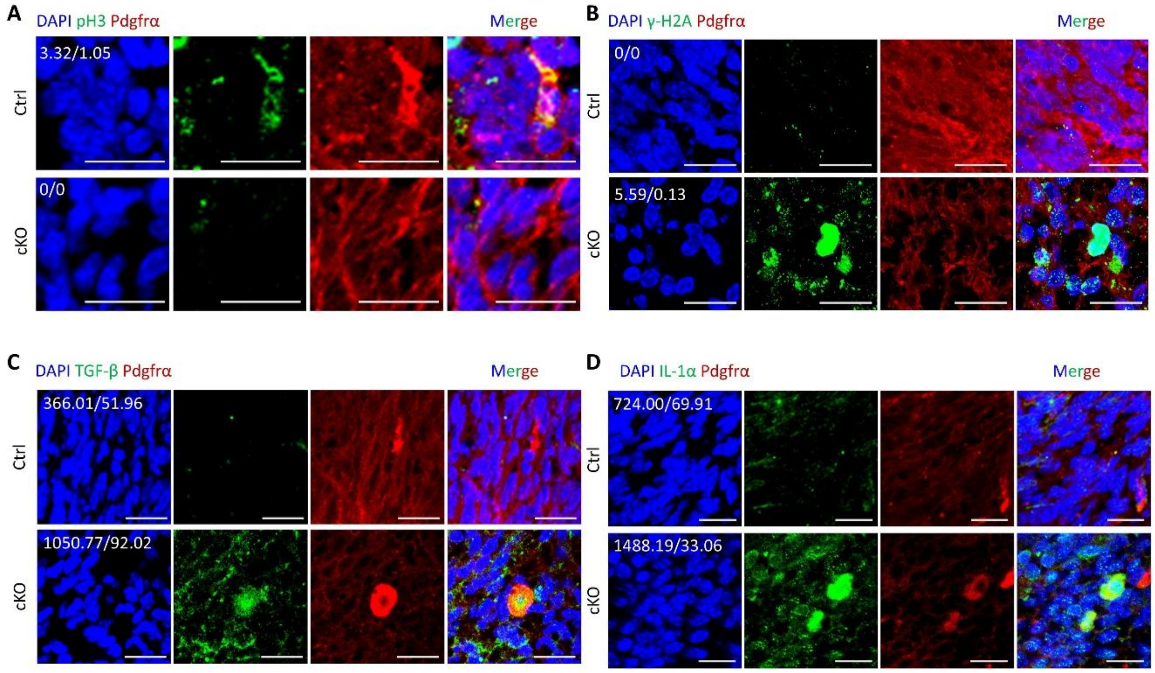

(A-D) Representative IF data of pH3, γH2A, TGF-β, and IL-1α, with the statistical results labelled (mean/SEM). *n* = 3 per group. Scale bars, 20 μm.

Figure S4. Extended data for senolytics therapy and dental injury models.

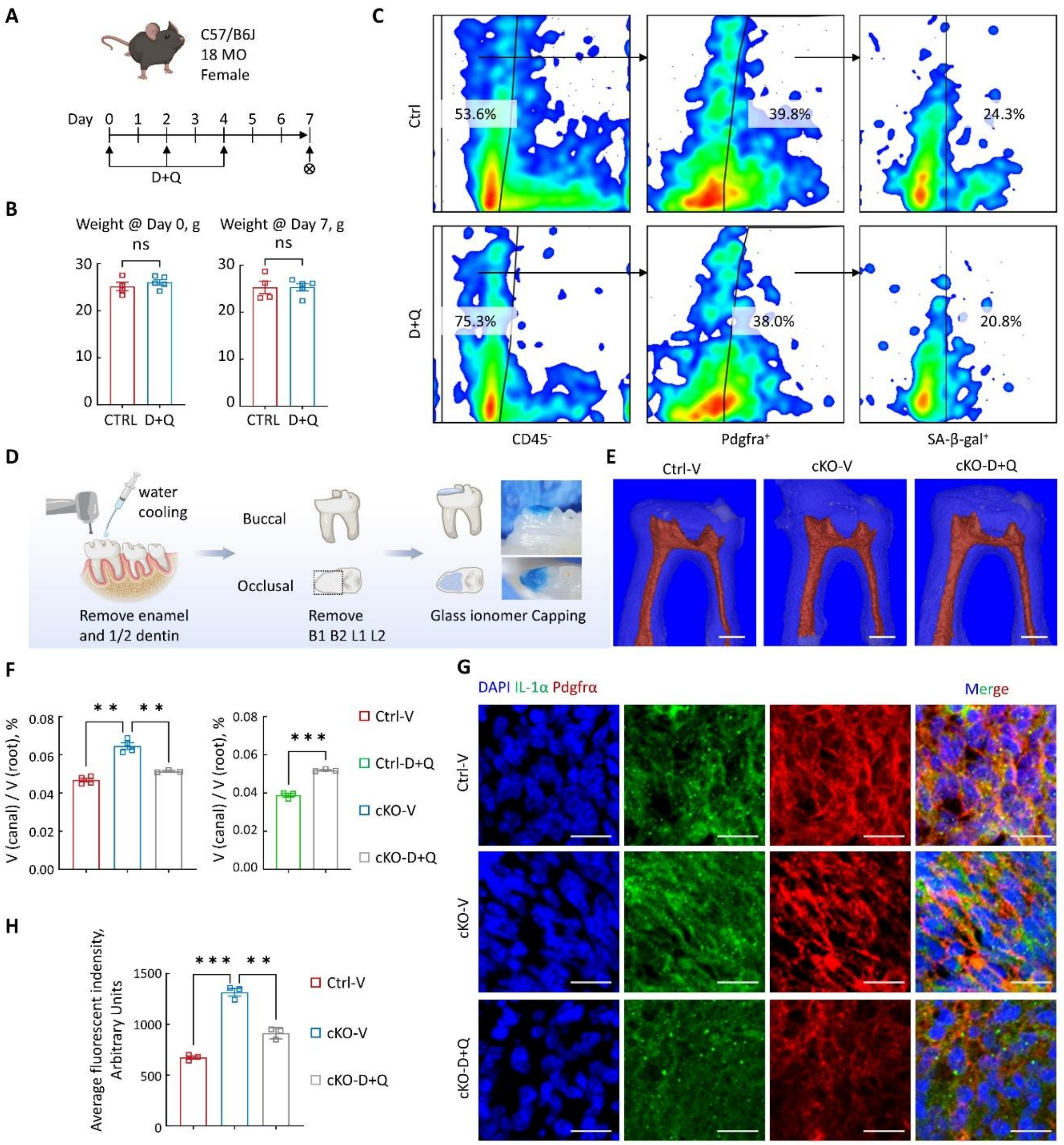

(A) Experimental timeline of validating the clearance efficiency of the D+Q treatment. (B) Body weight before senolytics administration and euthanasia, *n* = 4 for the control group and 5 for the D+Q group. (C) FACs workflow and gating hierarchy used to quantify $CD45^{-}Pdgfra^{+}SA\text{-}\beta\text{-}gal^{+}$ cells. (D) Schematic illustration for the dental injury model, referring to Fig. 5. B1, B2, L1, L2: the buccal and lingual cusps of the first molar. (E) Representative reconstruction images of dental pulp volume and the total root volume, referring to Fig. 5J-K. Scale bars,500 μm. (F) Statistical data of (E). For the Ctrl-V and cKO-V groups, *n* = 4; for the Ctrl-D+Q and cKO-D+Q group, *n* = 3. (G-H) Representative IF images of IL-1α (G) and their statistical results (H), For the Ctrl-V and cKO-V groups, *n* = 4; for the cKO-D+Q group, *n* = 3. Scale bars, 20 μm.

** $p < 0.01$, *** $p < 0.005$, ^{ns} no significance

Figure S5. Detailed procedures of tissue-clearing based double labelling analyses for murine molars.

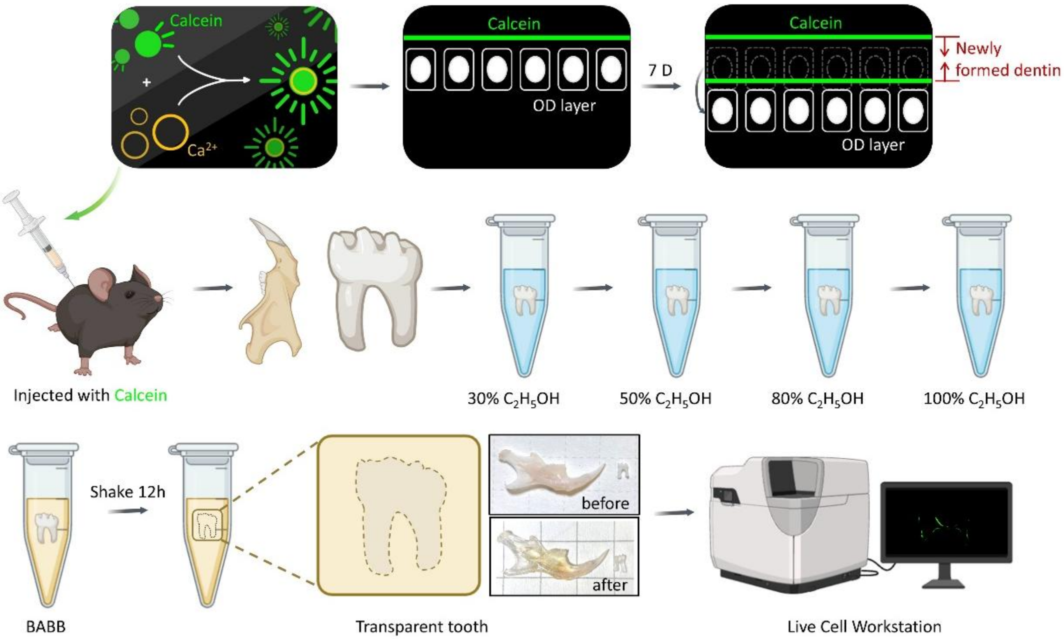

BABB, the transparent working solution was prepared by mixing benzyl benzoate (Sigma-Aldrich) and benzyl alcohol (Sigma-Aldrich) in a 2:1 volume ratio, referring to Fig. 5D.

SUPPLEMENTAL TABLES

Table S1. Characteristics of enrolled cases by group.

	Young	Aged
Population (Female/Male)	55/29	25/14
Age (Min-Max, Mean±SEM)	18-40, 28.12±0.63	60-87, 70.79±1.27
Arch (Maxillary/Mandibular)	66/57	51/58
Position (Anterior/Posterior)	10/113	28/81
Outcome (IP/Pulp survival)	12/111	34/75

Table S2. Summary of the association between age and irreversible pulpitis progression.

Model	Covariate adjustment	Age effect estimate (OR, 95%CI)	*P* value
Crude	None	1.0215 (1.0060-1.0382)	0.0077
Multivariable	Sex, dental arch, and tooth position	1.0180 (1.0020-1.0360)	0.0330
Mixed-effects	Sex, dental arch, and tooth position; patient-level random intercept included	1.0403 (1.0383-1.0422)	< 0.001
Sensitivity	One tooth per patient retained, iterating 1000 times	Mean OR = 1.0294[*]	Median *p* = 0.0135

* OR > 1 in 100% of iterations and statistically significant in 95.3% of iterations.

Table S3. 108 core DnDEGs in human dental pulp MSC senescence.

Genes	Uniprot Annotation	Genes	Uniprot Annotation
MCM10	Nucleus	TROAP	cytosol
ORC1	Nucleus	MCM5	Cytosol
LMNB1	Nucleus	DLGAP5	Cytoskeleton
CDC45	Nucleus	RRM2	Cytosol
E2F8	Nucleus	PBK	Nucleus
CENPA	Nucleus	KNL1	Nucleus
MKI67	Nucleus	BUB1	Nucleus
SKA1	Cytoskeleton	HJURP	Nucleus
EXO1	Nucleus	ERCC6L	Cytosol
CDT1	Nucleus	CEP55	Cytoskeleton
KIF18B	Cytoskeleton	CDCA5	cytosol
NCAPH	Cytoskeleton	CDCA8	Cytoskeleton
PLK4	Cytoskeleton	EZH2	Nucleus
GINS1	Nucleus	TPX2	Cytoskeleton
AURKB	Cytoskeleton	KIF15	Cytoskeleton
SPC24	Nucleus	KIF2C	Nucleus
SKA3	Cytoskeleton	ESPL1	Cytoskeleton
NCAPG	Nucleus	SPAG5	Cytoskeleton
NUF2	Nucleus	NEK2	Cytoskeleton
MCM2	Nucleus	CENPM	Cytoskeleton
PLK1	Cytoskeleton	TTK	Cytoskeleton
SPC25	Nucleus	RAD54L	Nucleus
GTSE1	Cytoskeleton	ZNF367	Nucleus
BUB1B	Cytoskeleton	TACC3	Cytoskeleton
KIF11	Cytoskeleton	DEPDC1	Nucleus
ASPM	Cytoskeleton	CENPW	Nucleus
WDR76	Nucleus	CENPF	Cytoskeleton
UBE2C	Cytosol	NEIL3	Nucleus
CDC20	Cytoskeleton	CCNA2	Nucleus
UHRF1	Nucleus	SHCBP1	Cytoskeleton
CCNF	Cytoskeleton	DEPDC1B	Cytosol
NDC80	Nucleus	KIF4A	Cytoskeleton
TCF19	Nucleus	NUSAP1	Cytoskeleton
RAD51AP1	Nucleus	MAD2L1	Cytoskeleton
ZWINT	Nucleus	UBE2T	Nucleus
TRIP13	Nucleus	CDKN2C	Cytoskeleton
MND1	Nucleus	CHTF18	Nucleus
RACGAP1	Plasma membrane	APOBEC3B	Nucleus
FAM83D	Cytoskeleton	CKS1B	Nucleus
SOX11	Nucleus	CDKN3	Cytosol

TYMS	Nucleus	KIF20A	Golgi apparatus
PRC1	Cytoskeleton	SMC4	Nucleus
SGO2	Nucleus	CENPH	Nucleus
HMMR	Plasma membrane	MELK	Plasma membrane
CCNB2	Cytoskeleton	GPSM2	Plasma membrane
TOP2A	Nucleus	TK1	Cytosol
ANLN	Cytoskeleton	GAS2L3	Cytoskeleton
CDCA3	Cytosol	AURKA	Cytoskeleton
KIF14	Cytoskeleton	PRR11	Nucleus
NCAPG2	Nucleus	POC1A	Cytoskeleton
CCNB1	Nucleus	KIF23	Cytoskeleton
OIP5	Cytoskeleton	BRIP1	Nucleus
BIRC5	Cytoskeleton	PSRC1	Cytoskeleton
TMPO	Nucleus	BARD1	Nucleus

45

Table S4. RT-qPCR primers.

Gene		Sequence (5’-3’)
GAPDH	Forward	GCTCTCTGCTCCTCCTGTTCG
	Reverse	GCGAACACATCCGGCCTGC
NFATC1	Forward	GCATCACAGGGAAGACCGTGTC
	Reverse	GAAGTTCAATGTCGGAGTTTCTGAG
ALPL	Forward	GACCTCCTCGGAAGACACTC
	Reverse	TGAAGGGCTTCTTGTCTGTG
COL1A1	Forward	TCTAGACATGTTCAGCTTTGTGGAC
	Reverse	TCTGTACGCAGGTGATTGGTG
SP7	Forward	TCTCCATCTGCCTGACTCCT
	Reverse	AGCGTATGGCTTCTTTGTGC
IBSP	Forward	CAGGCCACGATATTATCTTTACA
	Reverse	CTCCTCTTCTTCCTCCTCCTC
SPP1	Forward	ATGATGGCCGAGGTGATAGT
	Reverse	ACCATTCAACTCCTCGCTTT
TNFRSF11B	Forward	GTGTGCGAATGCAAGGAAGG
	Reverse	CCACTCCAAATCCAGGAGGG
DSPP	Forward	CAACCATAGAGAAAGCAAACGCG
	Reverse	TTTCTGTTGCCACTGCTGGGAC
