# Supplementary material for: NFATC1 dysfunction-triggered MSC senescence induces tooth aging amenable to senolytic therapy
Source: Stem Cell Reports. 2026 May 21;21(6):102925. doi: 10.1016/j.stemcr.2026.102925 (PMC13261951; doi:10.1016/j.stemcr.2026.102925)
Supplement: Document S2. Article plus supplemental information [file mmc4.pdf]

STEM CELL REPORTS
Article
OPEN ACCESS

NFATC1 dysfunction-triggered MSC senescence induces tooth aging amenable to senolytic therapy

Feifei Li,[1,2,5] Changhao Yu,[1,3,5] Lin Yao,[1,4,5] Yawen Tang,[1,2,5] Xue Yang,[1,3] Yitian Wang,[1] Jianxin Liu,[1,3] Bei Yin,[1,3] Haisheng Wang,[1] and Fanyuan Yu[1,3,6,*]
[1]State Key Laboratory of Oral Diseases & National Center for Stomatology & National Clinical Research Center for Oral Diseases, West China Hospital of Stomatology, Sichuan University, Chengdu, China
[2]Department of Pediatric Dentistry, West China Hospital of Stomatology, Sichuan University, Chengdu, China
[3]Department of Endodontics, West China Hospital of Stomatology, Sichuan University, Chengdu, China
[4]Department of Dentistry, The 4th West China Hospital, Sichuan University, Chengdu, China
[5]These authors contributed equally
[6]Lead contact
*Correspondence: fanyuan_yu@outlook.com
https://doi.org/10.1016/j.stemcr.2026.102925

SUMMARY

Organ-specific aging drivers extend our understanding of aging and offer therapeutic potential for combating age-related decline and rejuvenating organ function. Mature mammalian teeth possess unique characteristics, cell-free calcified parenchyma, isolated vasculature, specialized metabolic environment, limited turnover, and replenishment of repair-associated cell lineages, distinguishing them from other organs and leaving tooth aging mechanisms largely unexplored. Here, by analyzing clinical data from human tooth aging and developing genetic tools, comprising Cre-based pulse-chase tracing and ablation, gene manipulation combined with tracing, and fluorescent ubiquitination-based cell cycle indicator (FUCCI), we identify the first *in vivo* driver of tooth aging. We further demonstrate that this driver induces senescence in dental pulp mesenchymal stromal cells (MSCs), mechanistically explaining irreversible organ degeneration and regenerative disability during aging. Moreover, senolytic therapy effectively ameliorates phenotypic alterations of tooth aging caused by disfunction of this driver and restores dental repair capacity. Our findings elucidate mechanisms of tooth aging and provide promising strategies for tooth preservation during aging.

INTRODUCTION

Unlike soft organs, mammalian teeth possess a cell-free parenchyma composed of mineralized enamel and dentin once fully formed. Consequently, the dental pulp—the tooth's mesenchyme—is the sole tissue communicating with the body via vasculature and nerves (Arola et al., 2017; Gronthos et al., 2000; Kaukua et al., 2014; Ketterl, 1983; Maeda, 2020). The hard chamber formed by enamel and dentin protects the centrally located pulp but simultaneously isolates it, resulting in uniquely low pulp turnover (Arola et al., 2017; Gronthos et al., 2000; Kaukua et al., 2014; Ketterl, 1983; Maeda, 2020). Dental pulp parameters (size, cell number, and metabolic activity) are orders of magnitude smaller than those of bone marrow. Each tooth contains a finite number of odontoblasts (ODs), the exclusive source of post-developmental dentinogenesis, unlike the constantly replenished osteogenic lineages in bone (Arola et al., 2017; Gronthos et al., 2000; Shen et al., 2019). Due to these specialized characteristics, dental pulp cells undergo limited replicative senescence but exhibit predominant chronological senescence *in vivo*, making dental pulp an ideal model for studying chronological stromal aging. The *in vivo* drivers of tooth aging remain unknown (Maeda, 2020), yet clinically, tooth aging profoundly impacts dental function, disease development, and treatment outcomes (Maeda, 2020). Identifying these drivers is therefore critically important.

Although tooth aging is causally linked to age-associated dental degeneration and regenerative disability, its pathogenesis remains largely unelucidated, despite extensive documentation of its phenotypic alterations (Maeda, 2020). Over time, alongside senescence of the mineralized parenchyma, dental pulp undergoes irreversible changes impairing its renewal capacity. This leads to brittle teeth prone to fracture and susceptible to damage as pulpal degeneration progresses and dentinogenesis fails (Maeda, 2020). These age-related issues remain unresolved due to the unknown drivers. Recognition of this problem in dentistry is relatively recent, dating back only two decades (Maeda, 2020). There is now growing consensus on the importance of developing methods to counteract tooth aging, particularly pulp aging, as a crucial strategy for tooth conservation (Maeda, 2020). To address this gap, we established *in vivo* genetic tools comprising Cre-based pulse-chase tracing, Cre-based tracing and ablation, gene manipulation combined with tracing, and gene manipulation combined with FUCCI (Yang et al., 2024; Yu et al., 2022; Zhao et al., 2024). Combining these tools with tissue clearing (Yang et al., 2024), advanced 3D imaging (Yang et al., 2024), and serial histological and molecular analyses, we identified and validated the *in vivo* driver of tooth aging.

Published by Elsevier Inc. on behalf of International Society for Stem Cell Research.
This is an open access article under the CC BY license (http://creativecommons.org/licenses/by/4.0/).

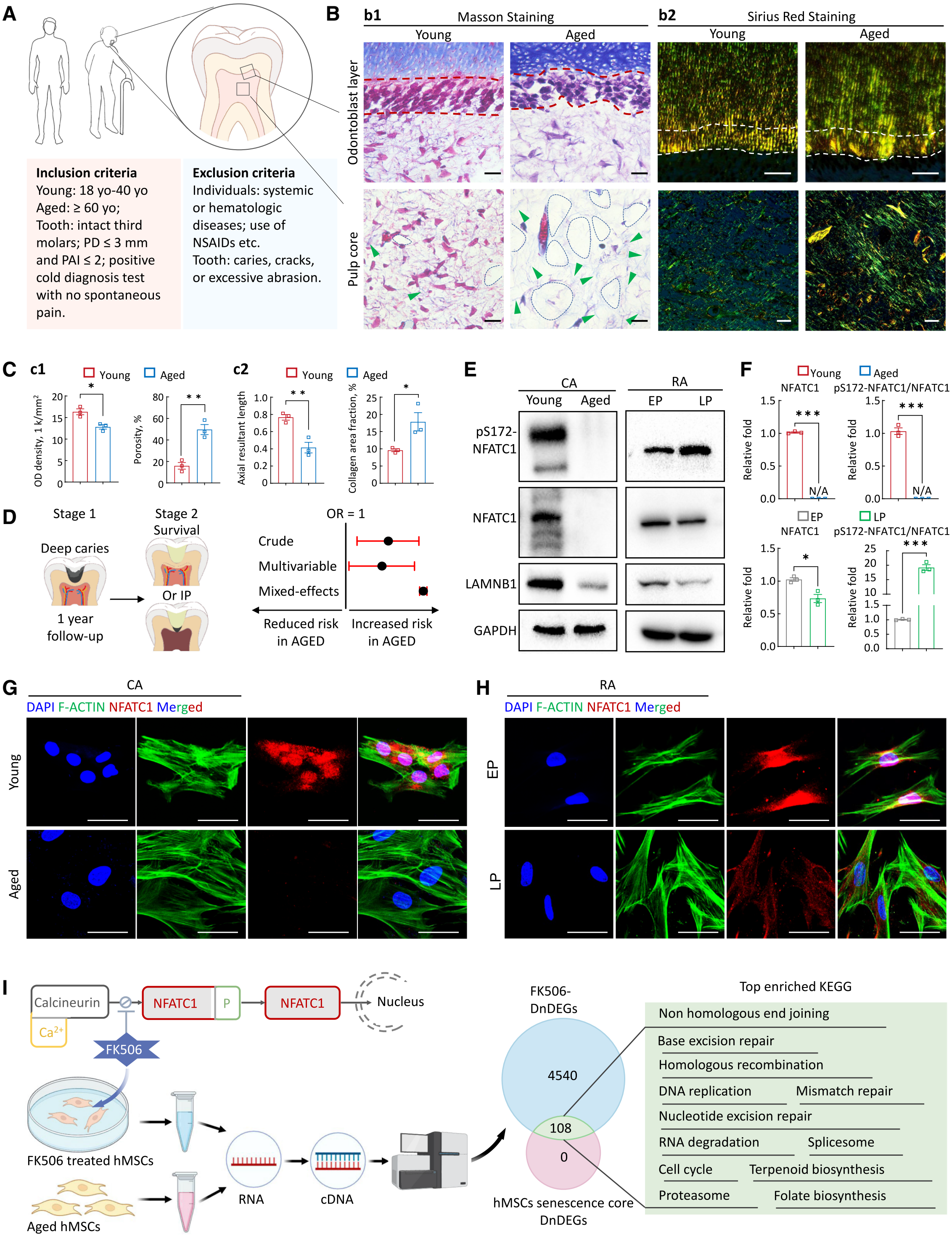

Figure 1. Identifying *NFATC1* dysfunction as the driver of human tooth aging

(A) Schematic summary of clinical analyses, regions of interest for (B), and the inclusion and criteria for human teeth samples. yo, years old.

(B) Representative Masson's trichrome (b1) and polarized light observed Sirius Red (b2) staining images of human third molars. Scale bars, 50 μm.

(C) Statistical analyses of Masson's trichrome (c1) and Sirius Red (c2) staining.

(legend continued on next page)

This driver accelerated aging, causing premature tooth aging in young adult mice. Mechanistically, we confirmed it as the cause of age-associated pulpal degeneration and regenerative disability. Crucially, we demonstrated that senolytics therapeutically ameliorate pulpal degeneration and restore regenerative capacity to preserve vital teeth by eliminating driver-induced senescent dental pulp MSCs. Similar to reports showing senescent skeletal stem cells create an inflammatory, degenerative environment impairing skeletal repair in aged bone (Ambrosi et al., 2021), our findings demonstrate that driver-induced dental pulp MSC senescence underlies poor regenerative activity in aging teeth.

RESULTS

Identifying NFATC1 dysfunction as a driver of human tooth aging

To uncover potential drivers of human tooth aging, we recruited young (18–40 years) and aged (≥60 years) systematically healthy volunteers of both genders and harvested their healthy third molars (Figure 1A). Masson's trichrome staining revealed that aged human dental pulp exhibited decreased cell density, depolarized ODs, disrupted fence-like ODs layer structures, and increased intercellular porosity and crosslinked fibers (Figures 1b1 and c1). Sirius red staining with polarized microscopy further showed increased collagen fibers in pulp cores and more depolarized predentin of aged teeth compared to young teeth (Figures 1b2 and c2). After characterizing histological changes, we analyzed regeneration-associated defense capabilities between groups. A case-control study (Figure 1D; Tables S1 and S2) enrolled teeth with clinically diagnosed deep caries followed for 1 year to determine irreversible pulpitis (IR) incidence rates. Clinical data showed a statistically significant odds ratio (OR) for IR in the aged group compared to the young group under three logistic regression models, indicating that age was a robust factor for higher risk of progression to IR. These evidence-based data confirm that tooth aging impairs dental repair, hindering the protective capacity to generate new dentin and pulp tissue against infection.

Having documented the pattern and functional deficiency, we investigated candidate drivers. Our prior studies revealed the critical role of *NFATC1*$^{+}$ MSCs in pulp formation and dentinogenesis (Yang et al., 2024), identified this population as *Pdgfra*^{high} (Yang et al., 2024), and linked *Pdgfra*^{high} cells to tooth aging (Ambrosi et al., 2021; Yao et al., 2023). Based on this, we hypothesized the involvement of *NFATC1* in human dental aging. We first measured *NFATC1* levels and activity during aging (Figures 1E and 1F). In aged *Pdgfra*^{high} dental MSCs exhibiting senescence (substantially reduced LAMNB1), *NFATC1* protein (pan or S172-phosphorylated/inactivated form) was undetectable (Figure 1E). Reduced pan *NFATC1* and increased inactivated form were also observed in replicative aging (RA, age-related decline that occurs as cells undergo repeated divisions) models (Figure 1E). Immunofluorescence (IF) confirmed *NFATC1* absence in *Pdgfra*^{high} pulp MSCs in chronologically aged (CA, age-related decline that occurs over time) samples compared to young controls (Figure 1F). IF for RA consistently showed substantially reduced total and nuclear (activated) *NFATC1* (Figures 1G and 1H). These data suggest *NFATC1* dysfunction (this term was used to denote impaired *NFATC1* activity) positively correlates with human dental pulp MSC senescence. Re-analysis of our RNA-seq data from human MSCs treated with FK506 (a *Ca^{2+}/NFATC1* inhibitor, which we validated induces *NFATC1* dysfunction) (Yu et al., 2022) using 108 core gene signatures of human dental pulp MSC senescence (Yu et al., 2023) (Figure 1I; Table S3) revealed that *NFATC1* dysfunction significantly downregulated all 108 signatures, including aging-associated genome instability and cell-cycle arrest (Yu et al., 2023) (Figure 1H). Furthermore, on interfering the *NFATC1* expression with siRNA, *in vitro* human dental pulp stem cells (hDPSC) showed a marked increase in the Ki67^{-}SA-β-gal^{+} senile fraction (Figure S1). These results led us to hypothesize that *NFATC1* dysfunction drives tooth aging, prompting the development of *in vivo* genetic tools for further analysis.

Ablation of NFATC1-expressing dental pulp MSCs phenocopies tooth aging

As a prerequisite for investigating *NFATC1* dysfunction as a driver, we characterized the *in vivo* expression pattern and

(D) Overview of the case-control study design and summarized forest plot of logistic regression analyses. IP, irreversible pulpitis; OR, odds ratio.
(E and F) Representative images (E) and quantification data (F) of WB. CA, chronological aging; RA, replicative aging; EP, early passage, that is the 3rd passage; LP, late passage, that is the 12th passage. $n = 3$ per group; N/A, not detected.
(G and H) Representative IF images of *Pdgfra*^{high} dental pulp MSC. Scale bars, 10 μm. CA, chronological aging; RA, replicative aging. EP, early passage; LP, late passage.
(I) Schematic illustration of work flowchart for RNA-seq. DnDEGs, down-regulated differentially expressed genes, $|\log_2\text{Foldchange}| > 0.5$, $p < 0.05$. KEGG, Kyoto Encyclopedia of Genes and Genomes, $Padj < 0.05$ was considered to be significantly enriched in KEGG. $*p < 0.05$, $**p < 0.01$, $***p < 0.005$ by Student's t test, error bars represent standard error of the data points. (C and F).

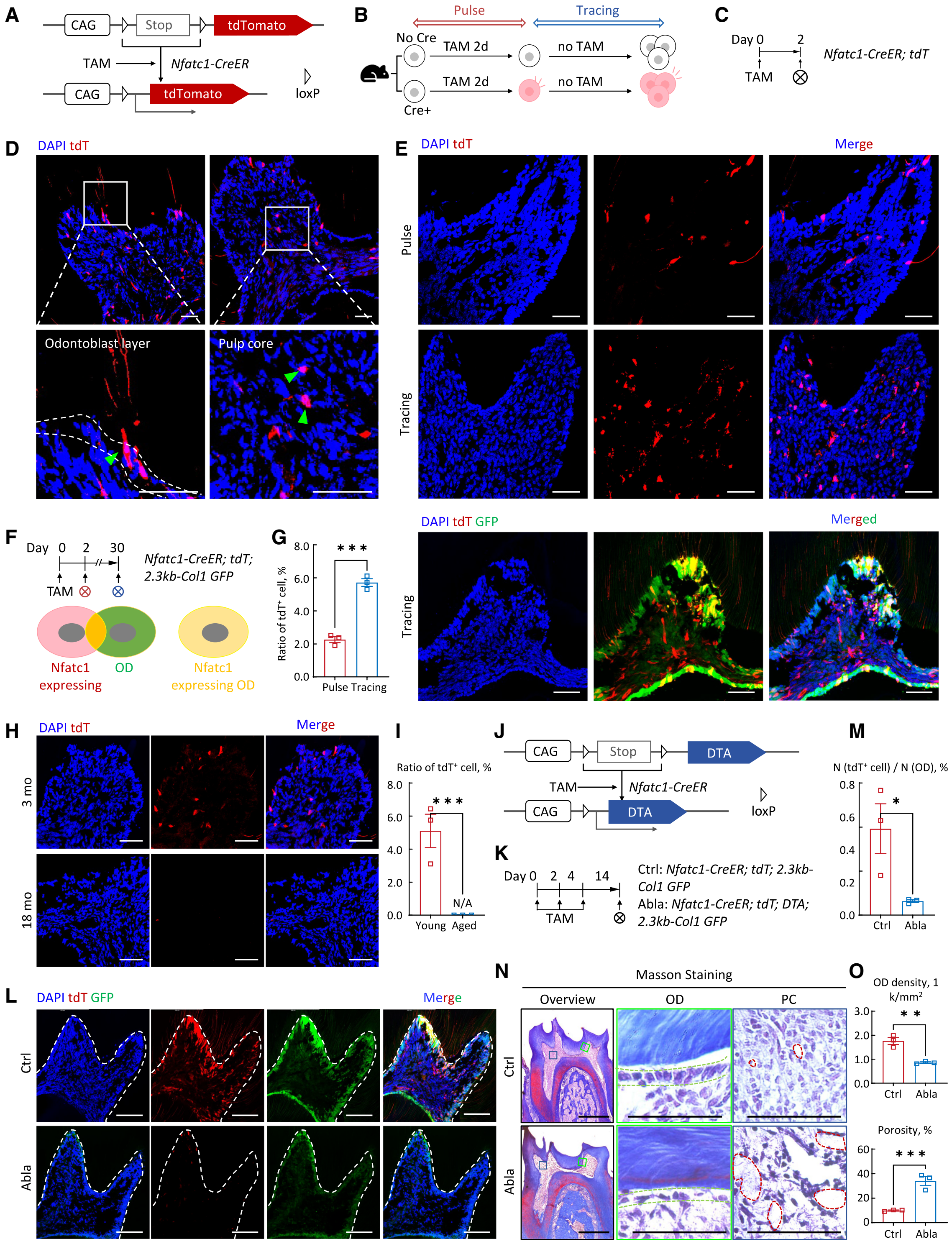

Figure 2. Ablation of *NFATC1*-expressing dental pulp MSCs phenocopies tooth aging

(A and B) Schematic illustration (A) and tracking readout (B) of the pulse-chase tracing genetic tool of $NFATC1^{+}$ cells. TAM, tamoxifen.
(C and D) Timeline (C) and representative fluorescent images (D) of pulse experiment of $NFATC1^{+}$ cells in 3MO mice's first mandibular molar. tdT, tdTomato. Dotted lines indicate the ODs layer, and the arrow heads indicate representative OD cell or pulp core MSC. Scale bars, 50 μm.

(legend continued on next page)

biological function of *NFATC1*$^+$ cells in teeth (Figure 2). Following our previous work (Yang et al., 2024; Yu et al., 2022), we generated a pulse-chase tracing model for *NFATC1*$^+$ cells (*Nfatc1-CreER; tdTomato* mice) (Figures 2A–2C). Pulse labeling in 3-month (mo) mice showed *NFATC1*$^+$ populations primarily in pulp core MSCs and minimally in ODs within molar pulp (Figure 2D), consistent with prior findings (Yang et al., 2024). Combining this with the odontoblastic reporter strain (*2.3kb-Col1 GFP*) (Figure 2F), we traced the *in vivo* trajectory of *NFATC1*$^+$ lineages (Figures 2E–2G). After 30 days (d) tracing, *tdTomato*$^+$ cells significantly increased in the pulp core compared to pulse-labeled controls (Figure 2E), statistically demonstrating continuous replenishment of pulp tissues by *NFATC1*$^+$ MSCs in adult teeth (Figure 2G). Tracing confirmed *NFATC1*$^+$ MSCs gave rise to *GFP*$^+$*tdTomato*$^+$ ODs, proving their role in generating pulp mesenchyme and new dentinogenic lineages. Notably, *NFATC1*$^+$ cells were remarkably decreased, becoming barely detectable in 18-month mice compared to 3-month mice (Figures 2H and 2I), reaffirming the *in vivo* link between *NFATC1* dysfunction in pulp MSCs and tooth aging.

We next established a Cre-based tracing and ablation tool to determine the functional role of *NFATC1*$^+$ cells in dental homeostasis (Figures 2J–2M). Ablation of *NFATC1*$^+$ MSCs significantly reduced proliferating *Pdgfra*^{high} pulp MSCs, indicating cell-cycle arrest (Figure S2A). Hallmarks of senescence-associated secretory phenotype (SASP), including IL-1α and TNF-α, were substantially upregulated post-ablation (Figures S2B and S2C), suggesting ablation induces tooth aging-like alterations. Combined with the ODs reporter (*2.3kb-Col1 GFP*), ablation severely hindered pulp core mesenchyme generation and nearly abolished odontogenic lineage formation (Figures 2L and 2M). Masson's trichrome staining showed that ablating *NFATC1*$^+$ MSCs in young teeth phenocopied age-related histological alterations: decreased pulp cell density, depolarized ODs, disrupted fence-like ODs layer, and increased intercellular porosity (Figures 2N and 2O). Together, these findings demonstrate that loss of *NFATC1*-expressing dental pulp MSCs in young adults induces tooth aging and impaired dentinogenesis.

Genetic induction of the tooth aging driver causes pulpal degeneration

To investigate whether *NFATC1* expression enables pulp MSCs to resist aging, we established a gene manipulation combined with tracing tool (Figures 3A and 3B) to track cell fate after knockout. We first confirmed efficient *NFATC1* deletion in pulp MSCs (Figure 3C). After 2 weeks (w) chase, conditional knockout (cKO) mice showed substantially fewer tdTomato$^+$ descendants in dental pulp, including reduced tdTomato$^+$ ODs and pulp core MSCs (Figures 3D and 3E). IF for the OD marker DSPP confirmed that *NFATC1* knockout in pulp MSCs completely halted ODs generation *in vivo* (Figures 3F and 3G). Lineage tracking indicated that genetically induced *NFATC1* dysfunction, mimicking its absence in aged human teeth (Figures 1F and 1G), impaired stromal genesis and odontogenesis.

H&E staining revealed thinner pre-dentin, decreased cell density, depolarized ODs, disrupted ODs layer, and increased intercellular porosity in cKO mice (Figure 3G). Inverted H&E images further highlighted increased porosity in cKO (Figure 3G). Masson's trichrome staining consistently demonstrated aging-like histological changes and detailed increased crosslinked fibers in cKO (Figure 3H). Quantitative analysis of H&E and Masson's trichrome data statistically confirmed that *NFATC1* cKO in pulp MSCs phenocopied tooth aging (Figures 3I and 3J). μCT analysis in 3-month mice further showed a significantly increased pulp volume/total root volume ratio in cKO mice (Figure 3L), indicating impaired dentinogenic capacity under homeostasis.

MSC senescence underlies NFATC1 dysfunction-driven tooth aging

As genetic *NFATC1* dysfunction phenocopied tooth aging, we investigated if aging occurs in cKO mice and the

(E and F) Representative fluorescent images (E) and schematic illustration (F) of the pulse-chasing data from *Nfatc1-CreER; tdTomato; 2.3kb-Col1 GFP* strain. Scale bars, 50 μm.
(G) Statistical analysis of (E), $n = 3$ per group.
(H and I) Representative fluorescent images of the pulse data from *Nfatc1-CreER; tdTomato*, respectively at 3 months and 18 months (H), and its statistical analyses (I). Scale bars, 50 μm. $n = 3$ per group.
(J) Schematic illustration of Cre-based ablation, which will be crossed with *tdTomato* reporter strain and *2.3kb-Col1 GFP* strain to get the Cre-based tracing and ablation tool.
(K–M) Experimental timeline (K), representative fluorescent images (L), and statistical analysis (M) for Cre-based tracing and ablation. Dotted lines in (L) contoured the ODs layer. Scale bars, 50 μm. OD in (M), *GFP*$^+$ ODs. $n = 3$ per group.
(N and O) Representative Masson's trichrome images (N) and statistical analyses (O) based on (K). Red dotted circles in (N) indicate the representative intercellular porosities; green dotted lines contoured the ODs layer. Scale bars: in low magnification, 500 μm; in high magnification, 100 μm. $n = 3$ per group in (O). $^*p < 0.05$, $^{**}p < 0.01$, $^{***}p < 0.005$ by Student's *t* test, error bars represent standard error of the data points. (G, I, M, and O).

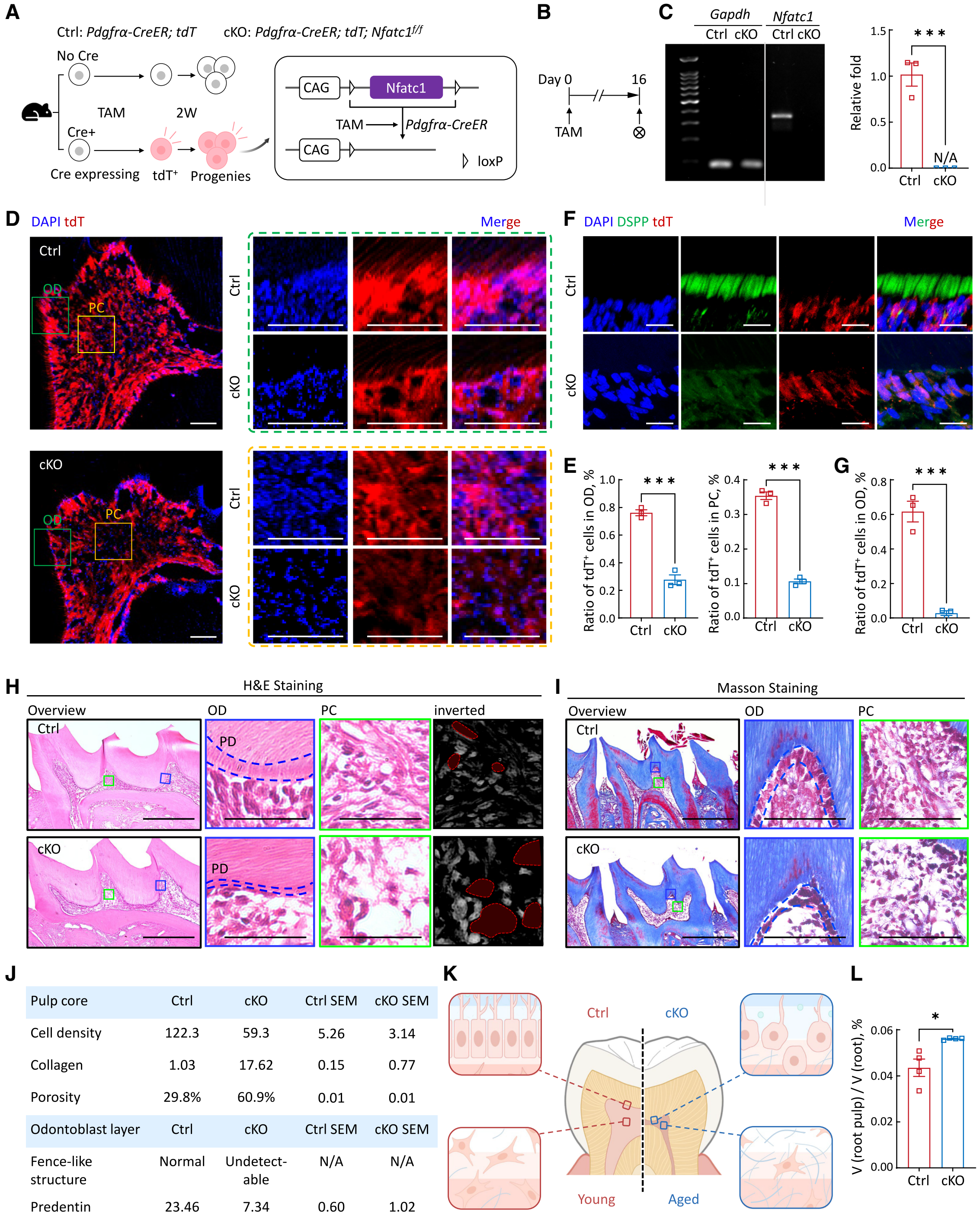

Pulp core	Ctrl	cKO	Ctrl SEM	cKO SEM
Cell density	122.3	59.3	5.26	3.14
Collagen	1.03	17.62	0.15	0.77
Porosity	29.8%	60.9%	0.01	0.01
**Odontoblast layer**	**Ctrl**	**cKO**	**Ctrl SEM**	**cKO SEM**
Fence-like structure	Normal	Undetect-able	N/A	N/A
Predentin	23.46	7.34	0.60	1.02

Figure 3. Genetically triggering tooth aging driver causes aging-associated degeneration

(A) Schematic illustration of the genetic tool of gene manipulation combined with tracing.

(B) Experimental timeline of the transgenic model in (A).

(C) Representative gel images (left) and statistical data (right) of quantitative RT-qPCR. N/A, undetectable. n = 3 per group.

(D and E) Representative fluorescent images (D) and statistical analyses (E) of (A) and (B). OD, odontoblast; PC, pulp core. Scale bars, 50 μm. n = 3 per group.

(legend continued on next page)

underlying mechanism. We developed FACS procedures specific to pulp MSCs in the gene manipulation combined with tracing model (Figures 4A and 4B). FACS of *CD45*$^{-}$ pulp MSCs showed reduced *tdTomato*$^{+}$ cells in cKO (Figure 4C), and senescence-associated β-galactosidase (SA-β-gal) staining revealed 54.0% *SA-β-gal*$^{+}$*tdTomato*$^{+}$ *CD45*$^{-}$ pulp MSCs in cKO versus 15.9% in controls (Ctrl) (Figure 4C). We then established a genetic tool combining cKO with *in vivo* cell cycle monitoring: gene manipulation combined with FUCCI (Ctrl: *Pdgfra-CreER; Fucci*/cKO: *Pdgfra-CreER; Nfatc1*$^{fl/fl}$*; Fucci*) (Figure 4D). Tamoxifen (TAM) induced simultaneous *tdTomato* labeling, *NFATC1* cKO, and FUCCI-based cell cycle reporting in cKO (Figures 4D and 4E). This approach revealed that *NFATC1* loss in *CD45*$^{-}$ pulp MSCs reduced S/G2-M phase proportions from 0.94% (Ctrl) to 0.36% (cKO) (Figure 4F). G1/S proportions decreased slightly, while G1 proportions significantly increased to 1.33% in cKO (0.80% in Ctrl) (Figure 4F), indicating severe cell-cycle arrest. IF for the mitotic marker phospho-histone H3 (Ser10, pH3) confirmed cycle arrest in cKO (Figure S3A), and γH2AX IF showed associated genomic instability pressure (Figure S3B). IF and quantification of SASP factors demonstrated an inflammatory and degenerative environment in cKO pulp MSCs (Figures 4G, 4H, S3C, and S3D). Thus, NFATC1 deficiency causes dental pulp MSC senescence, driving tooth aging in cKO mice.

We next assessed if *NFATC1* knockout directly impaired dentinogenesis (Figures 4I–4L). DSPP IF showed damaged dentinogenesis *in vivo*, with nearly no polarized DSPP^{high} ODs or organized ODs layer in cKO (Figure 4I). We harvested *CD45–Pdgfra*^{high} pulp MSCs from 18-year-old adults and induced mineralization using osteogenic medium (OM) (Figures 4J–4L). Von Kossa staining after 28 days OM revealed that FK506 or direct *NFATC1* silencing significantly impaired MSC mineralization capacity (Figure 4J). RT-qPCR confirmed that FK506-mediated NFATC1 inhibition significantly downregulated key early (*ALPL*, *COL1A1*, and *SP7*) and late (*IBSP*, *SPP1*, and *TNFRSF11B*) odontogenic genes (Figure 4K). Direct *NFATC1* silencing similarly downregulated these genes (Figure 4L), confirming *NFATC1* dysfunction as the cause of impaired odontogenesis.

Senolytics therapeutically ameliorate tooth aging-impaired dental regeneration

Having established *NFATC1* dysfunction drives tooth aging and impaired dentinogenesis, we tested its causal role in the regenerative disabilities observed clinically after dental injury (Figures 1C, S1B, and S1C). We established a dental injury model in young adult mice (Figure S4D), applied it to the gene manipulation combined with tracing strains (Figures 5A–5E), and incorporated double labeling, tissue clearing, and 3D imaging (Figures 5A–5E and S5). μCT at 14 days post-injury (dpi) showed significantly reduced reparative dentin and root dentin thickening in cKO mice (Figures 5B and 5C). 3D imaging of cleared, double-labeled samples provided stereoscopic evidence that NFATC1 knockout inhibited injury-responsive dentinogenesis (Figure 5D; Videos S1 and S2). Quantification of double labeling confirmed significantly reduced daily dentin regeneration in cKO (<0.5 MS/μm) versus Ctrl (1.5 MS/μm) (Figure 5E). Using the gene manipulation combined with FUCCI tool (Ctrl: *Pdgfra-CreER; Fucci*/cKO: *Pdgfra-CreER; Nfatc1*$^{fl/fl}$*; Fucci*) (Figure 5F), flow cytometry revealed NFATC1 cKO substantially reduced S/G2-M proportions (0.46% vs. Ctrl 0.83%) and increased G1 proportions (3.58% vs. Ctrl 2.77%) after injury (Figure 5G), indicating regenerative disability.

Given reports that senolytics ameliorate age-related degeneration and regenerative disability in other organs (Thoppil and Riabowol, 2019), we tested if they could rescue *NFATC1* dysfunction-induced tooth aging and poor regeneration (Figures 5H and 5I). We first evaluated the ability of senolytics to eliminate senescent cells in aged mice (Figures S4A–S4C). Using a "hit and run" intermittent dosing regimen, we observed a 14.4% reduction in the proportion of *SA-β-gal*$^{+}$ senescent cells within the *CD45–Pdgfra*$^{+}$ pulp MSCs. Subsequently, in our *in vivo* model of *NFATC1* dysfunction, μCT reconstruction (Figure S4E) and quantification (Figure S4F) at 14 dpi showed a significantly increased pulp canal/root volume ratio in vehicle-treated cKO mice (cKO-V) versus Ctrl-V, indicating impaired dentin formation. Senolytic treatment (Dasatinib plus Quercetin, D + Q) in cKO mice (cKO-D+Q) reduced this ratio to levels equivalent to Ctrl-V (Figures S4E and S4F). Focusing on reparative dentin regeneration, μCT confirmed D + Q

(F and G) Representative IF images of DSPP (F) and statistical analysis (G) based on (A) and (B). Scale bars, 50 μm. $n = 3$ per group.
(H–J) Representative H&E (H) and Masson's trichrome (I) images and statistical data (J) based on (A) and (B). Dotted lines contoured the pre-dentin (H) or the ODs layer (I). Scale bars: in low magnification, 500 μm; in high magnification, 50 μm. In the inverted images, the red bubbles indicated the representative images of intercellular porosities. $n = 3$ per group.
(K) Graphical summary of the identical phenotypic alterations between human tooth aging and the genetic tool of gene manipulation combined with tracing.
(L) Statistical data of μCT based on (A) and (B), $n = 4$ per group. *$p < 0.05$, ***$p < 0.005$ by Student's t test, error bars represent standard error of the data points. (C, E, G, and L).

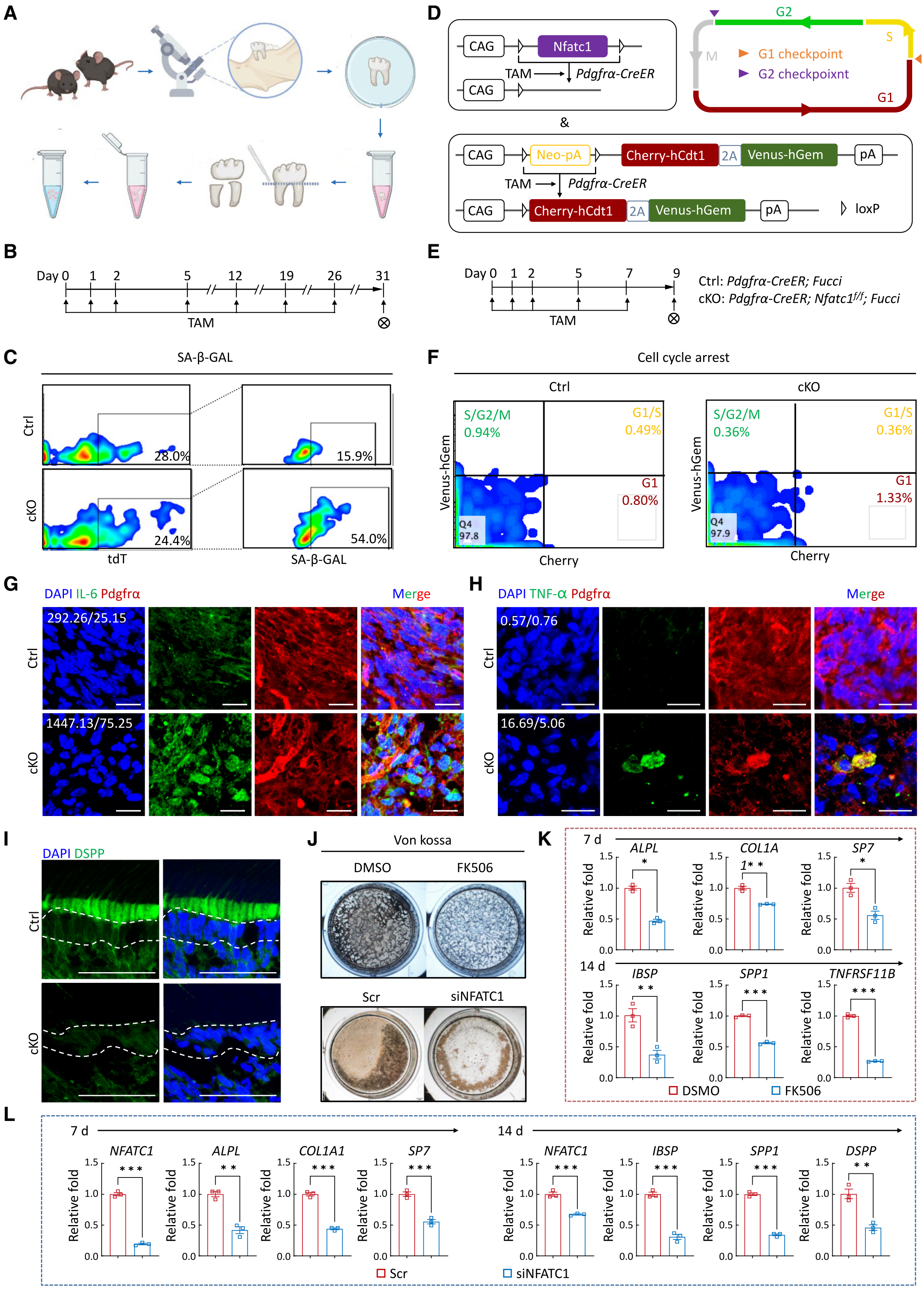

(legend on next page)

treatment effectively restored regenerative capacity in cKO mice to Ctrl-V levels by 14 dpi (Figures 5J and 5K). Histologically, Masson's trichrome at 5 dpi showed cKO reduced reparative dentin formation and induced aging-like changes (decreased pulp cell density and increased porosity), while D + Q restored reparative dentin and pulp density while reducing porosity (Figure 5M). IF for Ki67 and quantification confirmed D + Q alleviated cKO-induced proliferative inhibition (Figure 5N), and SASP IF demonstrated D + Q effectively remedied the inflammatory and degenerative environment caused by *NFATC1* cKO (Figures 5N, 5O,S2H, and S2I).

DISCUSSION

Identifying organ-specific aging drivers is a promising therapeutic strategy for addressing the growing crisis of aging and associated diseases (Gulen et al., 2023; Liu et al., 2023; Singh et al., 2023). However, the unique characteristics of teeth—including a fully mineralized parenchyma, low turnover, isolated vasculature, and specialized metabolic environment—have left the *in vivo* driver of tooth aging undiscovered (Carvalho and Lussi, 2017; Maeda, 2020). Here, by developing genetic tools (Cre-based pulse-chase tracing, Cre-based tracing and ablation, gene manipulation combined with tracing, and gene manipulation combined with FUCCI), we identified *NFATC1* dysfunction as the first *in vivo* driver of tooth aging. Our human data linked *NFATC1* dysfunction to aging, and genetic tools confirmed its causal role in tooth aging and associated regenerative disability. Mechanistically, we revealed that *NFATC1* dysfunction induces dental pulp MSC senescence, triggering tooth aging. Furthermore, we demonstrated that "D + Q" senolytic therapy ameliorates *NFATC1* dysfunction-induced tooth aging, therapeutically rejuvenating adult teeth to aid preservation.

A key point arising from this study is the definition of the *NFATC1* dysfunction. Based on our data, we operationally define *NFATC1* dysfunction as impaired *NFATC1* activation, indicated by reduced nuclear localization, increased inhibitory Ser172 phosphorylation, and, in more advanced settings, depletion of the total NFATC1 protein pool. Under this framework, CA and RA likely represent different stages of a shared process of progressive NFATC1 inactivation. In RA, inactive pS172-NFATc1 remains detectable while nuclear NFATC1 is reduced, suggesting functional inactivation with residual protein still present. Whereas in chronologically aged MSCs, NFATC1 becomes further depleted and falls below the detection threshold. Thus, the apparently distinct protein patterns in the two models are interpreted as different manifestations of the same underlying loss of *NFATC1* function rather than unrelated biological events.

Although *NFATC1* has not been extensively studied in organ aging, its role in hair follicle stem cell (HFSC) aging has been discussed (Keyes et al., 2013; Roy et al., 2024; Wang et al., 2016; Zhang et al., 2021). Some evidence links aberrant *NFATC1* activation to HFSC aging and quiescence, proposing anti-*NFATC1* therapy for rejuvenation (Keyes et al., 2013; Roy et al., 2024; Wang et al., 2016). Conversely, other studies show that loss of *NFATC1* causes HFSC niche escape and aging (Zhang et al., 2021). Our findings deposit tooth aging in this broader debate and favor the view that *NFATC1* dysfunction can be pathogenic in adult stromal compartments. In dental pulp, *NFATC1* dysfunction was associated with senescence in human samples and induced aging phenotypes in transgenic murine models, indicating *NFATC1* as an active determinant of tissue decline in this context.

Beyond damaging nutritive, sensory, and protective functions, tooth aging profoundly impairs dentin-pulp complex regeneration (Carvalho and Lussi, 2017; Maeda, 2020), though the mechanisms remained unclear. Our results elucidate the causal mechanism. *NFATC1* dysfunction in pulp MSC was associated with impaired cell-cycle progression, as indicated by FUCCI-based profiling and the NFATC1 interference transcriptomic profiling. This was accompanied by suppression of odontogenic differentiation-related programs, supported by decreased expression

Figure 4. MSCs senescence is the reason for *NFATC1* dysfunction-driven tooth aging

(A) Schematic illustration of the procedures prepared for FACs, including the molars harvest, dissection, and digestion to finally obtain single-cell suspension of dental pulp.

(B and C) Experimental timeline (B) and representative FACs results (C) of gene manipulation combined with tracing. Ctrl, *Pdgfra-CreER; tdTomato*. cKO, *Pdgfra-CreER; tdTomato; Nfatc1*$^{fl/fl}$. tdT, tdTomato.

(D–F) Schematic illustration (D), experimental timeline (E), and representative FACs results (F) of the gene manipulation combined with FUCCI, including the readout conditions.

(G–I) Representative IF data of IL-6 (G), TNF-α (H), and DSPP (I) based on (B), with the statistical data of mean/SEM provided on (G) and (H). Scale bars: 20 μm for (G) and (H) and 50 μm for (I).

(J) Von Kossa staining results at the 28 days after osteogenic induction of human dental pulp MSC. Scr, scrambled RNAs.

(K and L) Statistical data of RT-qPCR at the time point labeled after osteogenic induction of human dental pulp MSCs. Scr, scrambled RNAs. $n = 3$ per group. $^{*}p < 0.05$, $^{**}p < 0.01$, $^{***}p < 0.005$ by Student's t test, error bars represent standard error of the data points. (K).

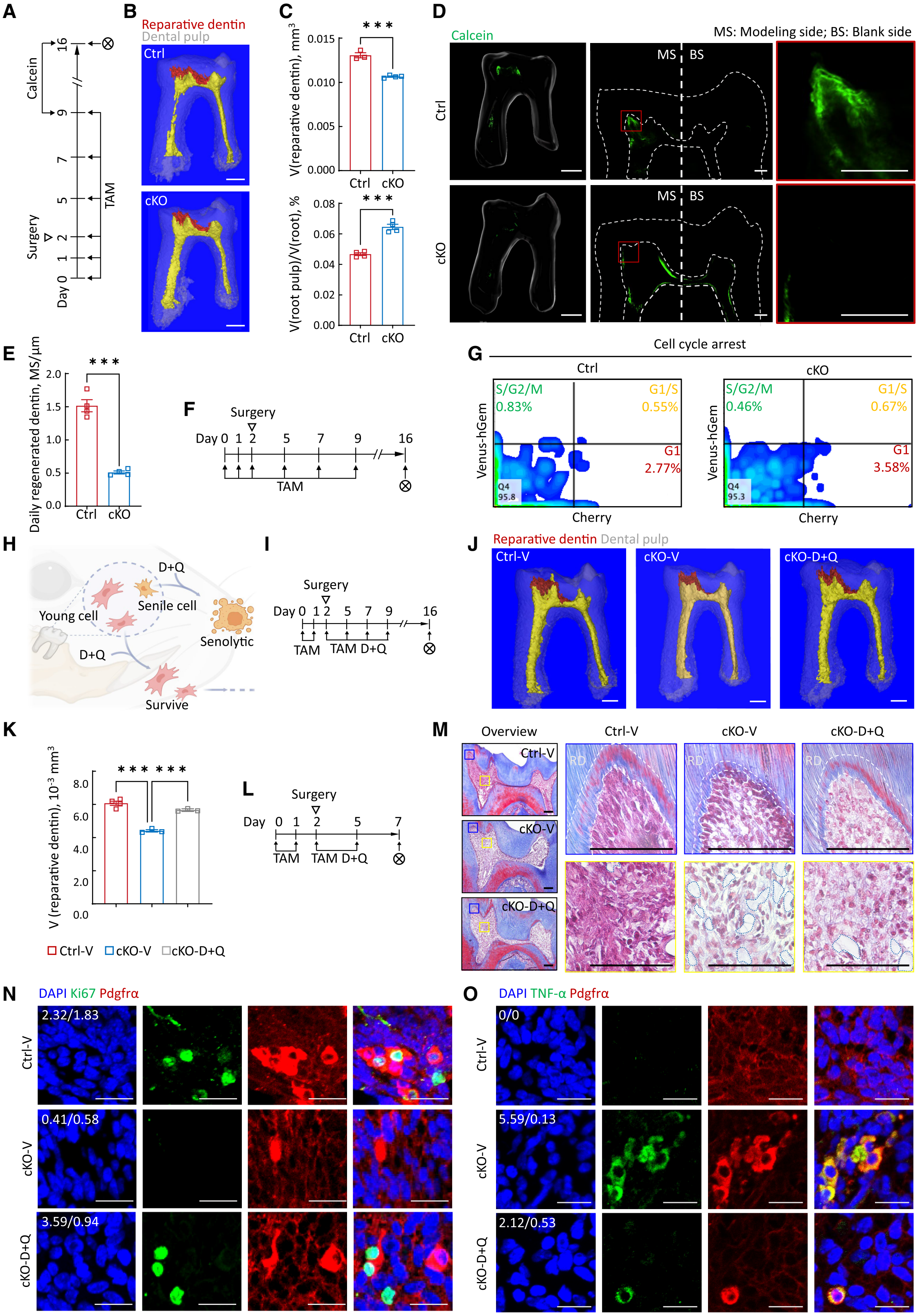

(legend on next page)

of mineralization-associated genes and impaired reparative dentin formation. Moreover, *NFATC1* dysfunction coincided with an elevated SASP-like features, including increased IL-1α, TGF-β, and TNF-α. Collectively, these findings suggest that *NFATC1* contributes to maintain a regenerative MSC state by supporting proliferative competence and odontogenic potential, while restraining SASP remodeling. Loss of this regulation may therefore shift the pulp microenvironment from repair-permissive to degeneration-prone.

However, several limitations of this study should be acknowledged. First, the pulp stromal populations analyzed here (including *Pdgfra*^{high} cells) yield heterogeneous, non-clonal cultures spanning a differentiation continuum, from stem subsets to lineage-committed progenitors and more differentiated stromal cells. Therefore, although our data indicate that *NFATC1* dysfunction is associated with reduced stem features, the genetic manipulations in this study were applied to the broader stromal compartment rather than an exclusively purified stem cell population. Contributions from non-stem stromal subsets therefore cannot be excluded.

Second, the *Pdgfra-CreER; Nfatc1*$^{fl/fl}$ model is not equivalent to natural CA. Rather, it provides an experimentally controlled system to evaluate whether NFATC1 dysfunction is sufficient to elicit senescence-associated change while minimizing confounding systemic variables that accompany natural aging. As such, this model should be viewed as demonstrating a causal contribution of *NFATC1* dysfunction to tooth aging-related phenotypes rather than fully recapitulating the full complexity of natural tooth aging.

In summary, by developing genetic tools combined with advanced tissue clearing and imaging, we reveal *NFATC1* dysfunction as the driver of tooth aging and associated poor dental repair. We demonstrate that *NFATC1* dysfunction-induced dental pulp MSC senescence mechanistically drives tooth aging and that senolytics effectively counteract this aging to restore pulp MSC regenerative capacity. These findings advance our understanding of tooth aging and provide potential strategies for combating age-related dental decline.

RESOURCE AVAILABILITY

Lead contact

Requests for further information and resources should be directed to and will be fulfilled by the lead contact, Fanyuan Yu (fanyuan_yu@outlook.com).

Materials availability

All unique/stable reagents generated in this study are available from Prof. Fanyuan Yu (fanyuan_yu@outlook.com) with a completed Materials Transfer Agreement.

Data and code availability

RNA-seq data have been deposited at Gene Expression Omnibus as GSE160273 and are publicly available as of the date of publication. The bright-field microscopy images, confocal microscopy images, original WB images, clinical data, and processed statistical datasets reported in this study will be shared by the lead contact upon request. This paper does not report original code. Any additional information required to reanalyze the data reported in this paper is available from the lead contact upon request.

ACKNOWLEDGMENTS

This work was supported by National Natural Science Foundation of China 82522021 (F.Y.), 82571084 (F.L.), and Sichuan Province Science and Technology Program 2025ZNSFSC0754 (F.L.) and 2025NSFJQ0071 (F.Y.).

Figure 5. Senolytics therapeutically ameliorate tooth aging-impaired dental regeneration

(A) Experimental timeline of gene manipulation combined with tracing, and then incorporated with double labeling. Surgical procedures were detailed in Figure S4D.

(B and C) Representative images of 3D-reconstructed μCT (B) and statistical analyses (C) following the experimental procedures in (A). Ctrl, *Pdgfra-CreER; tdTomato*. cKO, *Pdgfra-CreER; tdTomato; Nfatc1*$^{fl/fl}$. Scale bars, 500 μm. $n = 4$ per group.

(D and E) Representative 3D images of double labeling (D) and statistical analysis (E) after tissue clearing, detailed in Figure S5. MS, modeling side; BS, blank side. Scale bars: in low magnification, 500 μm; in medium and high magnification, 100 μm. $n = 4$ per group.

(F and G) Experimental timeline (F) and representative FACs results (G) of gene manipulation combined with FUCCI.

(H) Graphical illustration of the workflow of senolytics on remedying senescent dental pulp MSC.

(I) Experimental timeline of senolytics for the transgenic model of gene manipulation combined with tracing after receiving dental injuries, harvesting at 14 days post-injuries. Ctrl, *Pdgfra-CreER; tdTomato*. cKO, *Pdgfra-CreER; tdTomato; Nfatc1*$^{fl/fl}$.

(J and K) Representative images of 3D-reconstructed μCT (J) and statistical analyses (K) following the experimental procedures in (I). Scale bars, 500 μm. $n = 3$ per group.

(L) Experimental timeline of the senolytics for the transgenic model of gene manipulation combined with tracing after receiving dental injuries, harvesting at 5 days post-injuries.

(M) Representative images of Masson's trichrome staining based on (L). White dotted lines contour the reparative dentin (RD), and blue dotted circles indicate the intercellular porosities. Scale bars, 100 μm.

(N and O) Representative IF images for Ki67 (N) and TNF-α (O) with the statistical results provided in each group (mean/SEM) based on (L). $n = 3$ per group. Scale bars, 20 μm. $^{***}p < 0.005$ by Student's t test, error bars represent standard error of the data points. (C, E, and K).

AUTHOR CONTRIBUTIONS

F.Y. and F.L. conceived the study; F.L., C.Y., L.Y., Y.T., X.Y., Y.W., J.L., B.L., H.W., and B.Y. performed research; F.Y., F.L., and Y.T. analyzed data; F.L. and F.Y. wrote the manuscript.

DECLARATION OF INTERESTS

The authors declare no competing interests.

DECLARATION OF GENERATIVE AI AND AI-ASSISTED TECHNOLOGIES IN THE WRITING PROCESS

The authors declare no generative AI and AI-assisted technologies used.

STAR★METHODS

Detailed methods are provided in the online version of this paper and include the following:

- KEY RESOURCES TABLE
- EXPERIMENTAL MODEL AND STUDY PARTICIPANT DETAILS
  - Ethics statements
  - Human teeth samples
  - Case-controlled study design
  - Animals
  - Indirect dental capping model
  - *In vivo* murine studies
- METHOD DETAILS
  - Cell culture and passaging
  - Mineralization-inducing culture
  - RT-qPCR
  - *NFATC1* deletion verification
  - DNA gel electrophoresis
  - Bulk RNA sequencing and bioinformatics analysis
  - Western blotting
  - Mouse pulp cell extraction and flow cytometry
  - Prepare tissue sections
  - Immunofluorescence staining
  - Histopathological staining and quantification
  - Mouse teeth clearing
  - μCT assessment
- QUANTIFICATION AND STATISTICAL ANALYSIS
  - General principle
  - Randomization and blinding
  - μCT analysis
  - Immunofluorescence quantification
  - Histopathological quantification

SUPPLEMENTAL INFORMATION

Supplemental information can be found online at https://doi.org/10.1016/j.stemcr.2026.102925.

Received: November 7, 2025
Revised: April 17, 2026
Accepted: April 19, 2026
Published: May 21, 2026

REFERENCES

Ambrosi, T.H., Marecic, O., McArdle, A., Sinha, R., Gulati, G.S., Tong, X., Wang, Y., Steininger, H.M., Hoover, M.Y., Koepke, L.S., et al. (2021). Aged skeletal stem cells generate an inflammatory degenerative niche. Nature *597*, 256–262. https://doi.org/10.1038/s41586-021-03795-7.

Arola, D.D., Gao, S., Zhang, H., and Masri, R. (2017). The Tooth: Its Structure and Properties. Dent. Clin. North Am. *61*, 651–668. https://doi.org/10.1016/j.cden.2017.05.001.

Carvalho, T.S., and Lussi, A. (2017). Age-related morphological, histological and functional changes in teeth. J. Oral Rehabil. *44*, 291–298. https://doi.org/10.1111/joor.12474.

Gonzalez Lopez, M., Huteckova, B., Lavicky, J., Zezula, N., Rakultsev, V., Fridrichova, V., Tuaima, H., Nottmeier, C., Petersen, J., Kavkova, M., et al. (2023). Spatiotemporal monitoring of hard tissue development reveals unknown features of tooth and bone development. Sci. Adv. *9*, eadi0482. https://doi.org/10.1126/sciadv.adi0482.

Gronthos, S., Mankani, M., Brahim, J., Robey, P.G., and Shi, S. (2000). Postnatal human dental pulp stem cells (DPSCs) in vitro and in vivo. Proc. Natl. Acad. Sci. USA *97*, 13625–13630. https://doi.org/10.1073/pnas.240309797.

Gulen, M.F., Samson, N., Keller, A., Schwabenland, M., Liu, C., Glück, S., Thacker, V.V., Favre, L., Mangeat, B., Kroese, L.J., et al. (2023). cGAS-STING drives ageing-related inflammation and neurodegeneration. Nature *620*, 374–380. https://doi.org/10.1038/s41586-023-06373-1.

Kaukua, N., Shahidi, M.K., Konstantinidou, C., Dyachuk, V., Kaucka, M., Furlan, A., An, Z., Wang, L., Hultman, I., Ahrlund-Richter, L., et al. (2014). Glial origin of mesenchymal stem cells in a tooth model system. Nature *513*, 551–554. https://doi.org/10.1038/nature13536.

Ketterl, W. (1983). Age-induced changes in the teeth and their attachment apparatus. Int. Dent. J. *33*, 262–271.

Keyes, B.E., Segal, J.P., Heller, E., Lien, W.H., Chang, C.Y., Guo, X., Oristian, D.S., Zheng, D., and Fuchs, E. (2013). Nfatc1 orchestrates aging in hair follicle stem cells. Proc. Natl. Acad. Sci. USA *110*, E4950–E4959. https://doi.org/10.1073/pnas.1320301110.

Liu, X., Liu, Z., Wu, Z., Ren, J., Fan, Y., Sun, L., Cao, G., Niu, Y., Zhang, B., Ji, Q., et al. (2023). Resurrection of endogenous retroviruses during aging reinforces senescence. Cell *186*, 287–304.e26. https://doi.org/10.1016/j.cell.2022.12.017.

Maeda, H. (2020). Aging and Senescence of Dental Pulp and Hard Tissues of the Tooth. Front. Cell Dev. Biol. *8*, 605996. https://doi.org/10.3389/fcell.2020.605996.

Roy, S., Mehta, D., Paradkar, A., Chovatiya, G., and Waghmare, S.K. (2024). Dab2 (Disabled-2), an adaptor protein, regulates self-renewal of hair follicle stem cells. Commun. Biol. *7*, 525. https://doi.org/10.1038/s42003-024-06047-2.

Shen, W.C., Lai, Y.C., Li, L.H., Liao, K., Lai, H.C., Kao, S.Y., Wang, J., Chuong, C.M., and Hung, S.C. (2019). Methylation and PTEN activation in dental pulp mesenchymal stem cells promotes osteogenesis and reduces oncogenesis. Nat. Commun. *10*, 2226. https://doi.org/10.1038/s41467-019-10197-x.

Singh, P., Gollapalli, K., Mangiola, S., Schranner, D., Yusuf, M.A., Chamoli, M., Shi, S.L., Lopes Bastos, B., Nair, T., Riermeier, A., et al. (2023). Taurine deficiency as a driver of aging. Science *380*, eabn9257. https://doi.org/10.1126/science.abn9257.

Tang, Y., Yu, C., Li, F., Wang, H., and Ye, L. (2023). Establishment of a Murine Pulp Exposure Model with a Novel Mouth-Gag for Pulpitis Research. J. Vis. Exp. https://doi.org/10.3791/66016.

Thoppil, H., and Riabowol, K. (2019). Senolytics: A Translational Bridge Between Cellular Senescence and Organismal Aging. Front. Cell Dev. Biol. *7*, 367. https://doi.org/10.3389/fcell.2019.00367.

Wang, L., Siegenthaler, J.A., Dowell, R.D., and Yi, R. (2016). Foxc1 reinforces quiescence in self-renewing hair follicle stem cells. Science *351*, 613–617. https://doi.org/10.1126/science.aad5440.

Yang, X., Han, C., Yu, C., Zhou, B., Ye, L., Li, F., and Yu, F. (2024). Inclusive, exclusive and hierarchical atlas of NFATc1(+)/PDGFR-α(+) cells in dental and periodontal mesenchyme. eLife *13*, RP100173. https://doi.org/10.7554/eLife.100173.

Yao, L., Li, F., Yu, C., Wang, H., Wang, Y., Ye, L., and Yu, F. (2023). Chronological and Replicative Aging of CD51(+)/PDGFR-α(+) Pulp Stromal Cells. J. Dent. Res. *102*, 929–937. https://doi.org/10.1177/00220345231158038.

Yu, F., Li, F., Yu, P., Zhou, B., and Ye, L. (2022). Identification and characterization of NFATc1(+) skeletal stem cells in bone regeneration. Cell Rep. *41*, 111599. https://doi.org/10.1016/j.celrep.2022.111599.

Yu, F., Yao, L., Li, F., Wang, C., and Ye, L. (2023). Releasing YAP dysfunction-caused replicative toxicity rejuvenates mesenchymal stem cells. Aging Cell *22*, e13913. https://doi.org/10.1111/acel.13913.

Zhang, C., Wang, D., Wang, J., Wang, L., Qiu, W., Kume, T., Dowell, R., and Yi, R. (2021). Escape of hair follicle stem cells causes stem cell exhaustion during aging. Nat. Aging *1*, 889–903. https://doi.org/10.1038/s43587-021-00103-w.

Zhao, H., Liu, Z., Chen, H., Han, M., Zhang, M., Liu, K., Jin, H., Liu, X., Shi, M., Pu, W., et al. (2024). Identifying specific functional roles for senescence across cell types. Cell *187*, 7314–7334.e21. https://doi.org/10.1016/j.cell.2024.09.021.

STAR★METHODS

KEY RESOURCES TABLE

REAGENT or RESOURCE	SOURCE	IDENTIFIER
**Antibodies**		
Rabbit monoclonal anti-IL-6	Abcam	RRID: AB_2889391
Rabbit polyclonal anti-TNF-α	Abcam	RRID: AB_305641
Rabbit polyclonal anti-DSPP	Abcam	Cat# ab216892
Rabbit monoclonal anti-TGF-β	Abcam	RRID: AB_2893156
Rabbit monoclonal anti-IL-1α	Abcam	RRID: AB_2941899
Rabbit monoclonal anti-PH3	CST	Cat# 3377
Rabbit monoclonal anti-Phospho-Histone H2A.X, Ser139 (γ-H2A)	CST	Cat# 9718S
Actin-Tracker Green	Beyotime	Cat# C1033
Mouse monoclonal anti-Pdgfra, Clone#16A1	Santa Cruz	RRID: AB_626904
Rabbit polyclonal anti-Ki67	Abcam	RRID: AB_443209
Goat Anti-Rabbit IgG H&L (Alexa Fluor® 488)	Abcam	RRID: AB_2630356
Goat Anti-Mouse IgG H&L (Alexa Fluor® 488)	Abcam	RRID: AB_2576208
Goat Anti-Mouse IgG H&L (Alexa Fluor® 594)	Abcam	RRID: AB_2650601
Rabbit polyclonal anti-Phospho-NFATC1, Ser172 (pS172-NFATC1)	SAB	Cat# 12620
Mouse monoclonal anti-NFATC1, Clone#7A6	Santa Cruz	RRID: AB_2152503
Rabbit polyclonal anti-LAMNB1	Bioworld	RRID: AB_1662868
Rabbit polyclonal anti-GAPDH	SAB	Cat# 21612
PerCP/Cyanine5.5 anti-mouse CD45	Biolegend	Cat# 103131
**Biological samples**		
Human extracted third molars	This study (Department of oral and maxillofacial surgery, West China Hospital of Stomatology, Sichuan University)	IRB approval WCHSIRB-D-2022-112; informed consent obtained
**Chemicals, peptides, and recombinant proteins**		
Tamoxifen	Sigma-Aldrich	Cat#T5648
Quercetin	Selleck	Cat#S2391
Dasatinib	Selleck	Cat#S1021
Methylene blue	Biosharp	Cat#BL1137A
Calcein	Sigma-Aldrich	Cat#C0875
α-MEM	Gibco	Cat#12571500
Fetal bovine serum (FBS)	Gibco	Cat#10091155

(Continued on next page)

Continued

REAGENT or RESOURCE	SOURCE	IDENTIFIER
Penicillin-streptomycin (P/S)	Gibco	Cat#15140122
DPBS, Ca^{2+} and Mg^{2+} free	Boster	Cat#PYG14190
Dexamethasone	Sigma-Aldrich	Cat#D4902
β-glycerophosphate	Sigma-Aldrich	Cat#G9422
Ascorbic acid	Sigma-Aldrich	Cat#A4403
Dimethyl sulfoxide (DMSO)	Sigma-Aldrich	Cat#D8418
FK506	Selleck	Cat#S5003
Human NFATC1 siRNA	Invitrogen	Cat#HSS143101
Trypsin Protease	Hyclone	Cat#SV30037.01
Agarose Molecular Biology Grade	Invitrogen	Cat#17850
TAE	Solarbio	Cat#T1060
Gold view	Solarbio	Cat#G8140
100 bp DNA Ladder	Vazyme	Cat#MD104-01
Red blood cell lysis buff	Solarbio	Cat#R1010
Collagenase I	BioFroxx	Cat#1904GR001
Benzyl benzoate	Sigma-Aldrich	Cat#B6630
Benzyl alcohol	Sigma-Aldrich	Cat#108006
Aluminum oxide	Sigma-Aldrich	Cat#199443
Ethyl cinnamate	Sigma-Aldrich	Cat#243000
DAPI Staining Solution	Beyotime	Cat#C1005
Sucrose	Sigma-Aldrich	Cat#V900116
4% paraformaldehyde	Boster	Cat#AR1068
Triton X-100	Thermo Fisher	Cat#85111
Bovine Serum Albumin (BSA)	Sigma-Aldrich	Cat# SRE0096
OCT tissue-freezing medium	Leica	Cat#14020108926
**Critical commercial assays**		
Masson's Trichrome Staining Kit	Solarbio	Cat#G1340
Hematoxylin and Eosin Staining Kit	Solarbio	Cat#G1120
Modified Sirius Red Staining Kit	Solarbio	Cat#G1472
Calcium Staining Kit (Von Kossa Method)	Solarbio	Cat#G3282
HiScript III RT SuperMix for qPCR	Vazyme	Cat#R323-01
AceQ Universal SYBR qPCR Master Mix	Vazyme	Cat#Q511-02
PrimeScript® RT Kit	Takara Bio	Cat#RR037A
TRIzol Plus RNA Purification Kit	Invitrogen	Cat#12183555
Glass Ionomer Luting Cement, Easymix	3M, Ketac	Cat#56900

(*Continued on next page*)

Continued		
**REAGENT or RESOURCE**	**SOURCE**	**IDENTIFIER**
**Deposited data**		
RNA-seq data	This study	GEO: GSE160273
**Experimental models: Organisms/strains**		
Nfatc1-CreER	Prof. Bin Zhou, Center for Excellence in Molecular Cell Science, CAS	In the published paper of Yu et al. (Yu et al., 2022)
tdTomato	JAX Lab	IMSR_JAX:007909
DTA	JAX Lab	IMSR_JAX:009669
2.3kb-Col1 GFP	JAX Lab	IMSR_JAX:016241
Pdgfra-CreER	JAX Lab	IMSR_JAX:018280
Nfatc1$^{fl/+}$	JAX Lab	IMSR_JAX:002786
Fucci	RIKEN BioResource Research Center	IMSR_RBRC02892
C57/B6J	Gempharmatech Experimental Animals Company	C57/B6J
**Oligonucleotides**		
GAPDH qPCR primer (Forward)	This study	5′-CTCTCTGCTCCTCCTGTTCG -3′
GAPDH qPCR primer (Reverse)	This study	5′- GCGAACACATCCGGCCTGC -3′
NFATC1 qPCR primer (Forward)	This study	5′- GCATCACAGGGAAGACCGTGTC -3′
NFATC1 qPCR primer (Reverse)	This study	5′- GAAGTTCAATGTCGGAGTTTCTGAG -3′
ALPL qPCR primer (Forward)	This study	5′- GACCTCCTCGGAAGACACTC -3′
ALPL qPCR primer (Reverse)	This study	5′- TGAAGGGCTTCTTGTCTGTG -3′
COL1A1 qPCR primer (Forward)	This study	5′- TCTAGACATGTTCAGCTTTGTGGAC -3′
COL1A1 qPCR primer (Reverse)	This study	5′- TCTGTACGCAGGTGATTGGTG -3′
SP7 qPCR primer (Forward)	This study	5′- TCTCCATCTGCCTGACTCCT -3′
SP7 qPCR primer (Reverse)	This study	5′- AGCGTATGGCTTCTTTGTGC -3′
IBSP qPCR primer (Forward)	This study	5′- CAGGCCACGATATTATCTTTACA -3′
IBSP qPCR primer (Reverse)	This study	5′- CTCCTCTTCTTCCTCCTCCTC -3′
SPP1 qPCR primer (Forward)	This study	5′- ATGATGGCCGAGGTGATAGT -3′
SPP1 qPCR primer (Reverse)	This study	5′- ACCATTCAACTCCTCGCTTT -3′
TNFRSF11B qPCR primer (Forward)	This study	5′- GTGTGCGAATGCAAGGAAGG -3′
TNFRSF11B qPCR primer (Reverse)	This study	5′- CCACTCCAAATCCAGGAGGG -3′
DSPP qPCR primer (Forward)	This study	5′- CAACCATAGAGAAAGCAAACGCG -3′
DSPP qPCR primer (Reverse)	This study	5′- TTTCTGTTGCCACTGCTGGGAC -3′
**Software and algorithms**		
GraphPad Prism software v9.5.1	GraphPad	https://www.graphpad.com/scientific-software/prism/RRID: SCR_002798

(*Continued on next page*)

Continued

REAGENT or RESOURCE	SOURCE	IDENTIFIER
Fiji (ImageJ v1.54m)	ImageJ	https://imagej.net<br>RRID: SCR_002285
FlowJo v10.8.1	FlowJo	https://www.flowjo.com
Imaris v9.9.0	Oxford Instruments	https://imaris.oxinst.com/RRID: SCR_007370
Mimics v21.0	Materialise	https://www.materialise.com/en/healthcare/mimics/mimics-core<br>RRID: SCR_015802
R Project for Statistical Computing	R Core Team	https://www.r-project.org/RRID: SCR_001905

EXPERIMENTAL MODEL AND STUDY PARTICIPANT DETAILS

Ethics statements

All human pulp tissue and clinical data procedures were reviewed and approved by the Ethical Committees of the West China School of Stomatology, Sichuan University (WCHSIRB-D-2022-112). The animal protocol was approved by the Ethics Committees of West China School of Stomatology, Sichuan University, and its approved number was WCHSIRB-D-2022-201. All experiments strictly followed the ethical requirements of the Ethics Committees of West China School of Stomatology, Sichuan University. All animal studies conformed to ARRIVE (Animal Research: Reporting of *In Vivo* Experiments) guidelines.

Human teeth samples

The human teeth used in this study were collected from patients undergoing impacted wisdom tooth extraction at the Department of Oral and Maxillofacial Surgery, West China Hospital of Stomatology, Sichuan University, from January 2022 to December 2024. All patients signed an informed consent form, and the research protocol received approval from the Medical Ethics Committee of the hospital (WCHSIRB-D-2022-112).

The inclusion criteria for the samples were as follows: intact third molar tooth (no obvious caries, no cracks, or excessive abrasion) with periodontal probing depth (PD) of ≤3 mm, periapical index (PAI) score of ≤2, a positive cold diagnosis test and no history of spontaneous pain. The exclusion criteria included: (1) systemic diseases (such as diabetes, osteoporosis, autoimmune diseases, etc.); (2) hematologic diseases or active infectious diseases; and (3) use of NSAIDs, glucocorticoids, bisphosphonates, or antimetabolite drugs within the past three months. The age group for the youth category was 18–40 years, while the senior category included individuals aged 60 years and older. The surgically extracted teeth were immediately rinsed with pre-cooled saline at 4°C to remove superficial blood clots. They were then transferred to an antibiotic-containing α-MEM (Gibco) preservation solution (100 U/mL penicillin and 100 μg/mL streptomycin, Gibco) and stored on ice. The samples were transferred to the laboratory's clean bench within two hours after extraction for further processing. All procedures were conducted in strict accordance with the Helsinki Declaration's Code of Ethics for the Study of Human Biospecimens.

Case-controlled study design

This study employed a prospective case-control design to explore the effects of age-related factor on the defensive capacity of pulp-dentin complex. The data was sourced from the standardized electronic health record system of the Department of Endodontics at West China Dental Hospital of Sichuan University from January 2023 to January 2025. All patients were diagnosed and treated by an associate chief physician with over 10 years of clinical experience. The research plan was reviewed and approved by the hospital's Medical Ethics Committee (WCHSIRB-D-2022-112), which granted an exemption from the requirement for informed consent.

The case screening criteria are as follows: (1) clinical examinations included a positive cold test, absence of spontaneous pain, and negative on percussion; (2) deep carious lesions that reached the middle to deep layers of dentin but no pulp perforation, indicated by digital apical film; (3) individuals between 18 and 40 years old (the young group) or above 60 years old (the aged group). Records have the following features are excluded: (1) diameter of the peri-root transmission area greater than 2 mm (PAI ≥3); (2) grade II mobility or intrabony defects; (3) history of dental treatment or trauma

within the past 6 months (4) previous radiation therapy or systemic chemotherapy; (5) salivary gland disease; and (6) enamel hypoplasia or abnormal dentin development.

After including the cases, follow-up records for each patient were tracked within a 1-year period through the Hospital Information System to determine whether the affected teeth progressed to irreversible pulpitis. Cases with missing follow-up data for any reason were excluded from the analysis. R studio was used to calculate the odds ratio (OR) for the relationship between age and the outcome of irreversible pulpitis. A 95% confidence interval was set, and a *p*-value of less than 0.05 was considered statistically significant. The case selection process was conducted independently by two physicians (Y.W.T & C.H.Y.).

Animals

All animal protocols in this study were reviewed and approved by the Ethics Committee of the West China School of Stomatology (WCHSIRB-D-2022-201), Sichuan University. The relevant surgical operations were performed in accordance with the standardized operating procedures of the State Key Laboratory of Oral Diseases. The experimental animals were housed in the specific-pathogen free Experimental Animal Core at the West China Hospital, Sichuan University. The environmental parameters were controlled as follows: a constant temperature of 25 ± 1°C, relative humidity of 50 ± 10%, and a 12-h light/dark cycle. The bedding consisted of autoclaved corn cob granules, supplemented with sterilized cotton wool for nesting.

The mouse strains used in this study are as follows: *Nfatc1-CreER* mice were reported in our previous study (Yu et al., 2022). *Pdgfrα-CreER* (JAX#018280), *tdTomato* (JAX#007914), *DTA* (JAX#009669), *2.3kb-Col1 GFP* (JAX#013134) and *Nfatc1*$^{fl/+}$ (JAX#022786) were purchased from the Jackson Laboratory. The *Fucci2a* (abbreviated as *Fucci* in this study) mice were purchased from RIKEN BioResource Research Center (stock# RBRC06511) with signed MTA. C57/B6J wild-type adult mice were obtained from Shanghai Model Organisms Center. For breeding *Nfatc1*$^{fl/fl}$ homozygous mice, we strictly adhered to the official genetic operation guidelines of the Jackson Laboratory. Heterozygous *Nfatc1*$^{fl/+}$ female mice (C57/B6J genetic background) were paired with male mice of the same genotype, and homozygous offspring were identified through PCR genotyping. Unless otherwise stated, all adult mice involved in this study were 3 months old, and all mice in the control group were housed together with the experimental group mice. Within each experimental batch, sex was matched between control and experimental groups; however, sex distribution may have varied across batches.

Indirect dental capping model

Once the state of surgical anesthesia was confirmed by the toe-pinch test, the mice were positioned supine on a surgical platform maintained at a constant temperature of 37°C to ensure core body temperature stability. A special mouth gag (Tang et al., 2023) was used to keep the jaw stable and open throughout the procedure. The surgeon employed a high-speed dental handpiece fitted with a 0.06 mm spherical emery needle in the right hand while holding a 26 G needle in the left hand to continuously inject saline, cooling the operation area. An assistant fully exposed the left mandibular first molar (M1) using a micro-retractor and simultaneously utilized a negative pressure suction system to remove coolant and dental debris. The four functional cusps of the M1 crown (mesial buccal cusp B1, near mesial buccal cusp B2, mesial lingual cusp L1, and near mesial lingual cusp L2) were removed sequentially using a layered cutting method, ensuring that the cutting depth matched the bottom of the fossil groove on the occlusal surface. To prepare the pulp cover material, glass ionomer cement (3M, Ketac, Easymix) was mixed with a methylene blue (Biosharp) tracer. This prepared material was applied to the surface of the exposed dentin after air-drying, and allowed to cure chemically for 2 min. Immediately following the procedure, meloxicam analgesic was injected intraperitoneally (5 mg/kg, 10 μL/g), and the animals were then transferred to a resuscitation chamber maintained at a constant temperature of 30°C for continuous monitoring until recovery of the righting reflex.

In vivo murine studies

Tamoxifen (Sigma-Aldrich) was prepared in 35 mg/mL using corn oil and stored frozen at −20°C. This solution was administered via intraperitoneal injection at a dose of 70 mg/kg. Dasatinib (Selleck) was dissolved in DMSO (Sigma-Aldrich) to create a stock solution of 100 mg/mL, stored at −20°C and protected from light. Quercetin (Selleck) was freshly prepared in 100 mg/mL aliquots using DMSO, then dilute to 50 mg/mL by adding sterile PBS (Boster). For the D + Q combined senolytics therapy, the following volume ratio was used: 1 part dasatinib stock solution, 20 parts quercetin aliquots, and 59 parts corn oil. These components were mixed to form a homogeneous suspension by vortexing 30 s. Prior to injection, the suspension was warmed in a 37°C water bath for 5 min and was then administered intraperitoneally at a dose of 5 μL/g.

METHOD DETAILS

Cell culture and passaging

The intact crown pulp from the third molar of healthy individuals was collected, and primary dental pulp cells were isolated using the tissue block adherence method. These primary cells were then expanded and cultured in α-MEM (Gibco), supplemented with 10% FBS (Gibco) and 100 U/mL penicillin along with 100 μg/mL streptomycin (Gibco). The culture was maintained at a constant temperature of 37°C and 5% CO_2 under controlled humidity. Once the adherent cells reached 80% confluence, they were digested and passaged at a ratio of 1:3. The experimental groups were designated as the early passaging group (P3) and the late passaging group (P12), with three independent donor-derived cell lines used repeatedly in each group.

Mineralization-inducing culture

The classical osteogenic induction system was employed for the mineralization-inducing of hDPSCs. This system included 10 nM dexamethasone (Sigma-Aldrich), 10 mM sodium β-glycerophosphate (Sigma-Aldrich), and 50 μg/mL ascorbic acid (Sigma-Aldrich) in a basal medium. The induction solution was changed every 48 h over a period of 14 days. To assess the induction endpoint qualitatively, von Kossa staining (Solarbio) was performed to identify calcium nodule formation. Additionally, the mRNA expression levels of osteogenesis-related marker genes were evaluated using RT-qPCR.

RT-qPCR

RNA extraction was conducted using the TRIzol method (Invitrogen), followed by cDNA synthesis through reverse transcription with HiScript III RT SuperMix (Vazyme). RT-qPCR was then performed on the iCycler real-time detection system (BioRad). The target mRNA levels were normalized using the Gapdh gene as a reference.

NFATC1 deletion verification

Mandibles from the Cre-based *Pdgfra*-reporter mice and the *Nfatc1*-cKO *Pdgfra*-reporter mice were harvested, as the input of control and experimental groups. Then the tissues were cropped and digested to obtain cell suspensions. After staining by a fluorophore-conjugated CD45 antibody, the cells were sorted by FACS to get *CD45*$^{-}$ Cre-targeted *Pdgfra*$^{+}$ MSC. Then these cells were lysed to obtain RNA to perform RT-qPCR.

DNA gel electrophoresis

Nucleic acid electrophoresis analysis was conducted using a 1.5% agarose (Invitrogen) gel and a TAE (Solarbio) buffer system. The visualization of DNA fragments was by adding GoldView nucleic acid dye (Solarbio). A 100 bp DNA ladder (Vazyme) was used as the molecular weight standard during loading. The electrophoresis was performed at a constant voltage of 120 V for 30 min. Gel imaging was carried out using the Bio-Rad Gel Doc XR + system.

Bulk RNA sequencing and bioinformatics analysis

Total RNA was extracted from human pulp mesenchymal stem cells (TRIzol, Invitrogen). Following a quality inspection using the Agilent 2100 Bioanalyzer, the library construction and double-ended 150 bp sequencing were performed on the Illumina NovaSeq 6000 by Novogene. Gene ontology functional enrichment analysis was conducted using clusterProfiler software. The criteria for screening differentially expressed genes were set as $|\log2$ (Fold Change)$| \geq 1$, and the false discovery rate corrected *p*-value was required to be less than 0.05. Subcellular localization information was annotated using the Uniprot database. All RNA-seq data were previously reported by us (Yu et al., 2023), with the accession number of GSE160273 on GEO.

Western blotting

After washing the cells with PBS (Boster), the cells were lysed using a lysis buffer containing protease and phosphatase inhibitor cocktail (Thermo Fisher). Total protein was then extracted according to the manufacturer's instructions. The proteins were separated by sodium dodecyl sulfate–polyacrylamide gel electrophoresis and transferred to polyvinylidene fluoride membranes. The membranes were blocked with 5% skimmed milk powder for 1 h. They were then incubated with a primary antibody (diluted by 5% BSA with the ratio per manufacturer's recommendation) overnight at 4°C, followed by an incubation with horseradish peroxidase-conjugated secondary antibody for 1 h at room tempera-

ture. For detection, enhanced chemiluminescent substrate was used, and the resulting signals were quantified using Fiji (ImageJ software). The data are standardized using GAPDH as an internal control.

Mouse pulp cell extraction and flow cytometry

Isolating molars from the maxilla and mandibles of mice under the microscope, then the periodontal ligament on the root surface of each tooth was scraped, and a solution of 2 mg/mL type I collagenase (BioFroxx) was used for stepwise digestion as follows: (1) preliminary digestion for 5 min to remove residual soft tissue; (2) separating the crown from the root along the dental cervix to expose the pulp cavity; (3) continuing digestion for an additional 40 min, with cell suspensions collected every 10 min. The digestion was terminated using a medium containing 10% FBS (Gibco). After passing the cell suspension through a 50 μm cell sieve, the pulp cells were obtained by centrifugation. After erythrocyte lysis, the final cell suspension was resuspended in PBS for flow cytometry analysis. The raw flow cytometry data were analyzed using cell population lapping analysis with FlowJo v10.8 software.

Prepare tissue sections

Mandibles were fixed in 4% paraformaldehyde at 4°C for 24 h, and placed in a 12% EDTA decalcification solution for 7 days. To prepare frozen section samples, the decalcified tissues were immersed in a 30% sucrose solution overnight at 4°C. The OCT embedding agent (Leica) was then used for freezing and embedding, and 5 μm thick continuous sections were prepared using a cryostat. These sections were stored at −20°C until further staining. For paraffin section samples, the tissues were dehydrated and soaked in paraffin after decalcification. After embedding in paraffin, 5 μm thick sections were prepared and stored at 4°C until subsequent staining.

Immunofluorescence staining

Tissue sections were blocked in PBS containing 0.1% Triton X-100 (Boster) and 5% BSA (Sigma-Aldrich) for 20 min. They were then incubated with primary antibodies overnight at 4°C. The following day, the sections were washed with PBS and incubated with fluorescent secondary antibodies, including DAPI (Beyotime) for nuclear staining, at room temperature in the dark for 2 h. Finally, the sections were mounted using anti-fade mounting medium. For cell samples, after fixation with 4% paraformaldehyde for 2 h, the remaining steps followed the same immunofluorescence staining process as used for the tissue sections.

Histopathological staining and quantification

After deparaffinization with xylene and gradient hydration with ethanol, the tissue sections were stained using hematoxylin-eosin (Solarbio), Masson's three-color staining (Solarbio), and modified Sirius red staining (Solarbio), following the instructions provided with the respective commercial kits. Then a laminator was used to apply neutral gum to the samples.

Mouse teeth clearing

The tissue clearing protocol used in this study is based on the method developed by Marcos et al. (Gonzalez Lopez et al., 2023). The BABB transparent working solution was prepared by mixing benzyl alcohol (Sigma-Aldrich) and benzyl benzoate (Sigma-Aldrich) in a 1:2 volume ratio. This mixture was then combined with activated neutral alumina (Sigma-Aldrich) to achieve a final concentration of 0.25 g/mL. The working solution was finally prepared by allowing it to stand overnight and centrifuging it to remove the supernatant. After clearing, samples were placed in a specialized Petri dish containing ethyl cinnamate (Sigma-Aldrich). Imaging was conducted using a Leica rotary confocal microscope, and the original image data was reconstructed in three dimensions using the IMARIS software (Oxford Instruments).

μCT assessment

The mandibles of euthanized mice were harvested, fixed in 4% paraformaldehyde, and stored in 70% ethanol. The μCT scan was conducted using the Scanco Medical μCT 45 system, with key parameters set at a tube voltage of 55 kVp, a tube current of 145 μA, and a reconstruction threshold of 220 mg/cm^3.

QUANTIFICATION AND STATISTICAL ANALYSIS

General principle

All results were verified through independent triplicate experiments. Statistical charts and analyses were generated using GraphPad Prism version 9.5.1. Unless specified otherwise, the following statistical methods will be employed in this

study: (1) For comparisons between two independent samples, a two-tailed *t* test will be conducted. The normality of each independent sample's data will be assessed using a normality test. (2) For comparisons involving three or more independent samples, a two-tailed one-way ANOVA will be performed. Normality and homogeneity of variance will also be tested for each independent sample's data. The significance level for this study is set at $p < 0.05$, with *, ** and *** denote $p < 0.05$, $p < 0.01$ and $p < 0.005$, respectively. All numerical data will be presented as mean ± SEM, based on results from at least three independent experiments. All *n* values in statistical analyses represent independent biological replicates. Technical replicates were averaged within each biological replicate and were not treated as independent *n* values.

Randomization and blinding

Randomization and blinding were not applicable to the clinical component, as this study was a case-control design. Likewise, the animal experiments compared distinct genetically modified mouse lines, which precluded random assignment and group blinding. The downstream procedures (specimen processing, data acquisition, and quantitative analysis) were performed with randomization and blinding to minimize bias.

μCT analysis

Morphometric parameters such as bone volume fraction (BV/TV) and bone mineral density (BMD) were calculated using standard algorithms provided in the system's supporting software. MIMICS software (Materialise) was then employed for three-dimensional reconstruction. The 16-bit DICOM files were imported into MIMICS, and the Mask tool was used to segment the tooth and pulp cavity. Within this region, voxels with grayscale values >31,000 were defined as reparative dentin.

Immunofluorescence quantification

For the reign of interest definition, in a crown pulp section, the odontoblast layer was identified as the outermost cellular layer based on DAPI staining, and the remaining cellular area of the crown pulp was identified as the pulp core. Cell percentages were calculated by manual counting using predefined positive-cell calling criteria. In detail, nuclei (DAPI) overlapping with tdTomato signal were counted as $tdTomato^+$ cells. The same criterion was applied to quantify $DSPP^+$, $pH3^+$, γ-$H2A^+$, TNF-α^+, and $Ki67^+$ cells.

The mean fluorescent intensity was measured by Fiji (ImageJ v1.54m). Briefly, composite IF images were split into individual 12-bit channels. The target-protein channel was thresholded using the Threshold tool according to the Fiji user guide, and MFI was obtained using the Measure function.

Histopathological quantification

For Masson's trichrome staining, two quantitative analyses were performed in entire coronal pulp contained sagittal sections. For odontoblast density, the odontoblastic layer was delineated as a region of interest (ROI), the number of odontoblastic cells within the ROI was counted, and the cell density was calculated as cell number divided by the measured ROI area. For vacuolar degeneration, the total pulp core area was obtained as ROI, and vacuolated regions within this ROI were further segmented and measured. The vacuolar degeneration was defined as porosity, which was calculated as vacuolated area divided by total pulp core area.

For Sirius red staining, two quantitative analyses were also performed under polarized light. For predentin fiber organization analysis, the predentin zone located within 50 μm beneath the odontoblastic layer was selected as the measurement region. Fiber orientation distribution was analyzed using the OrientationJ plugin in Fiji, and the degree of alignment was expressed as the axial resultant length calculated from the orientation distribution. For pulp core fibrosis analysis, collagen-positive regions within the pulp core ROI were segmented and measured, and the fibrotic area fraction was calculated as collagen-positive area divided by total pulp core area.

Stem Cell Reports, Volume *21*

Supplemental Information

NFATC1 dysfunction-triggered MSC senescence induces tooth aging amenable to senolytic therapy

Feifei Li, Changhao Yu, Lin Yao, Yawen Tang, Xue Yang, Yitian Wang, Jianxin Liu, Bei Yin, Haisheng Wang, and Fanyuan Yu

SUPPLEMENTAL FIGURES

Figure S1. Validation of *NFATC1* knockdown in promoting senescence of hDPC.

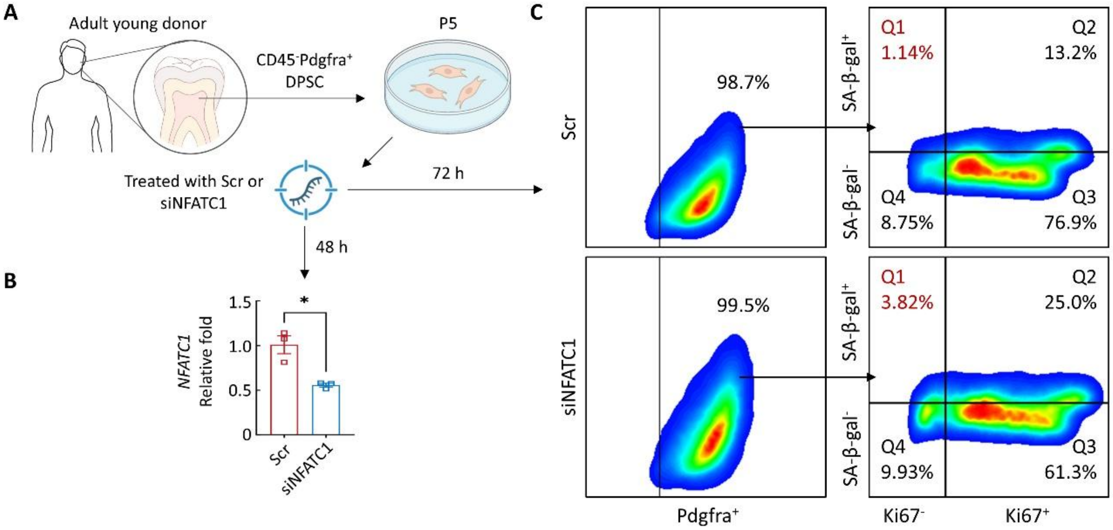

(A) Experimental workflow for siRNA-mediated NFATC1 knockdown in hDPSC. (B) Validation of siNFATC1 knockdown efficiency by RT-qPCR. (C) Flow cytometric analysis of senescent cell fraction following *NFATC1* knockdown.

* $p < 0.05$

Figure S2. Extended data for NFATC1-expressing dental pulp MSCs ablation models.

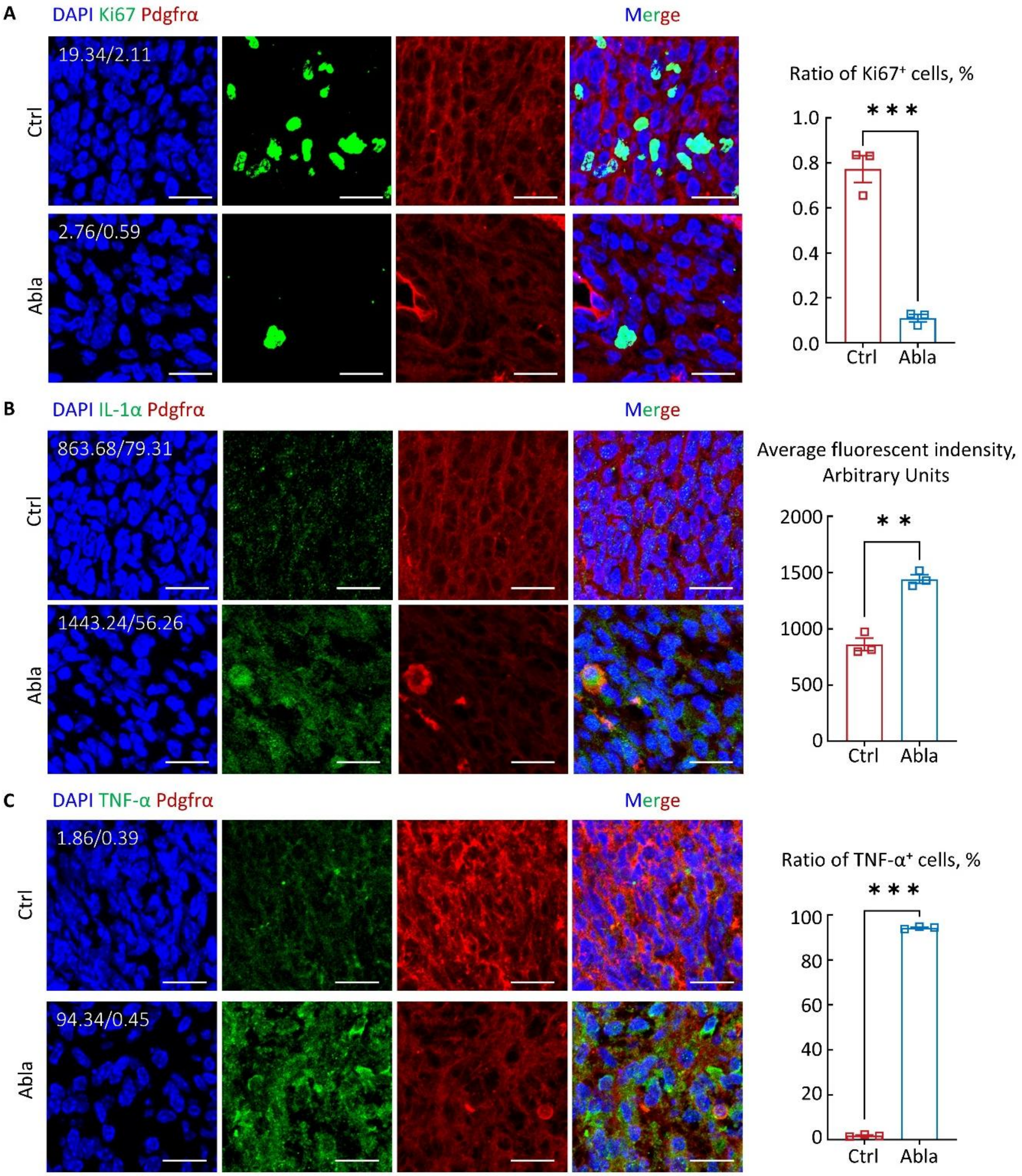

(A-C) Representative IF images of tracing and ablation, detecting the levels of Ki67, TNF-α, and Il-1α. The statistical data of IF were provided in each panel with mean/SEM. Scale bars, 200 μm. $n = 3$ per group.

** $p < 0.01$, *** $p < 0.005$

Figure S3. Extended data for the gene manipulation combined with tracing model.

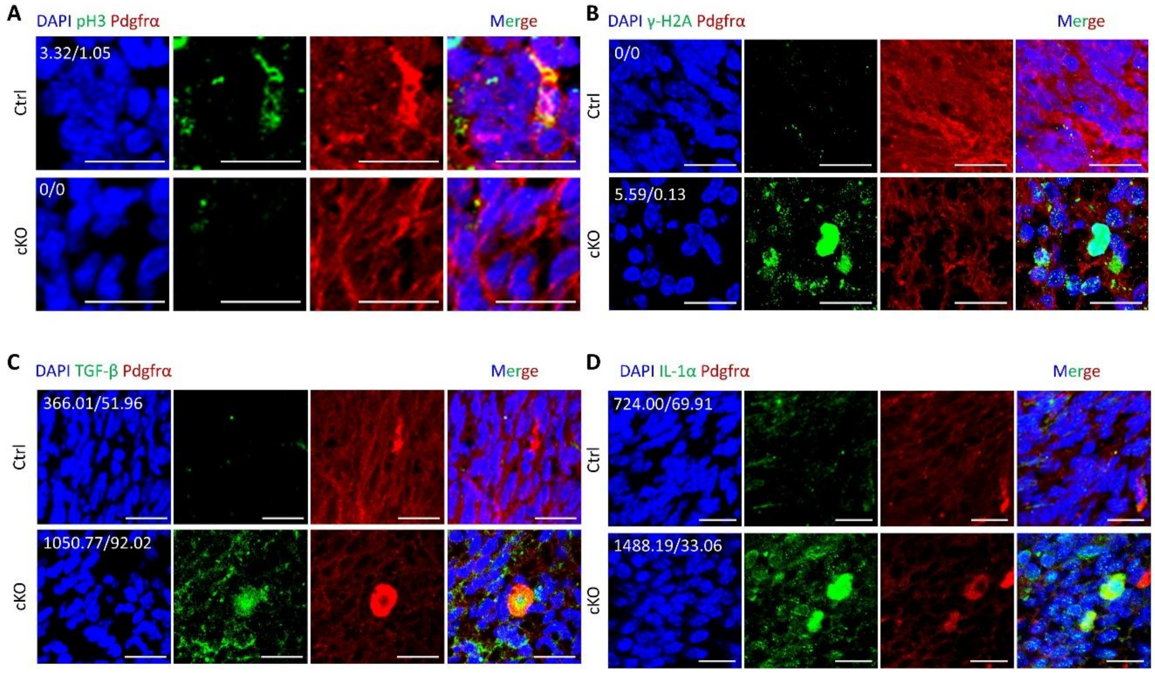

(A-D) Representative IF data of pH3, γH2A, TGF-β, and IL-1α, with the statistical results labelled (mean/SEM). *n* = 3 per group. Scale bars, 20 μm.

Figure S4. Extended data for senolytics therapy and dental injury models.

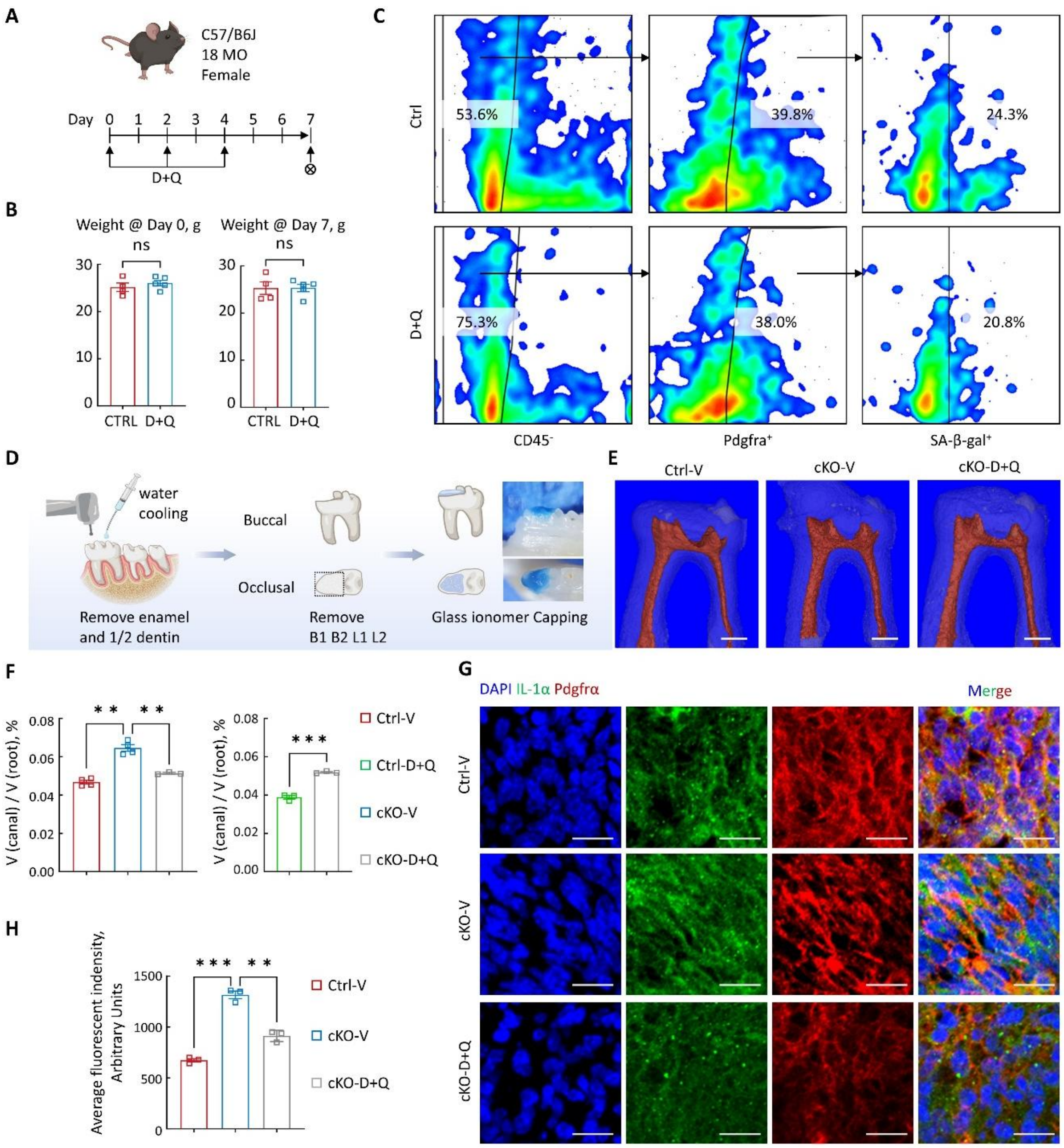

(A) Experimental timeline of validating the clearance efficiency of the D+Q treatment. (B) Body weight before senolytics administration and euthanasia, *n* = 4 for the control group and 5 for the D+Q group. (C) FACs workflow and gating hierarchy used to quantify *CD45⁻Pdgfra⁺SA-β-gal⁺* cells. (D) Schematic illustration for the dental injury model, referring to Fig. 5. B1, B2, L1, L2: the buccal and lingual cusps of the first molar. (E) Representative reconstruction images of dental pulp volume and the total root volume, referring to Fig. 5J-K. Scale bars,500 μm. (F) Statistical data of (E). For the Ctrl-V and cKO-V groups, *n* = 4; for the Ctrl-D+Q and cKO-D+Q group, *n* = 3. (G-H) Representative IF images of IL-1α (G) and their statistical results (H), For the Ctrl-V and cKO-V groups, *n* = 4; for the cKO-D+Q group, *n* = 3. Scale bars, 20 μm.

** $p < 0.01$, *** $p < 0.005$, ns no significance

Figure S5. Detailed procedures of tissue-clearing based double labelling analyses for murine molars.

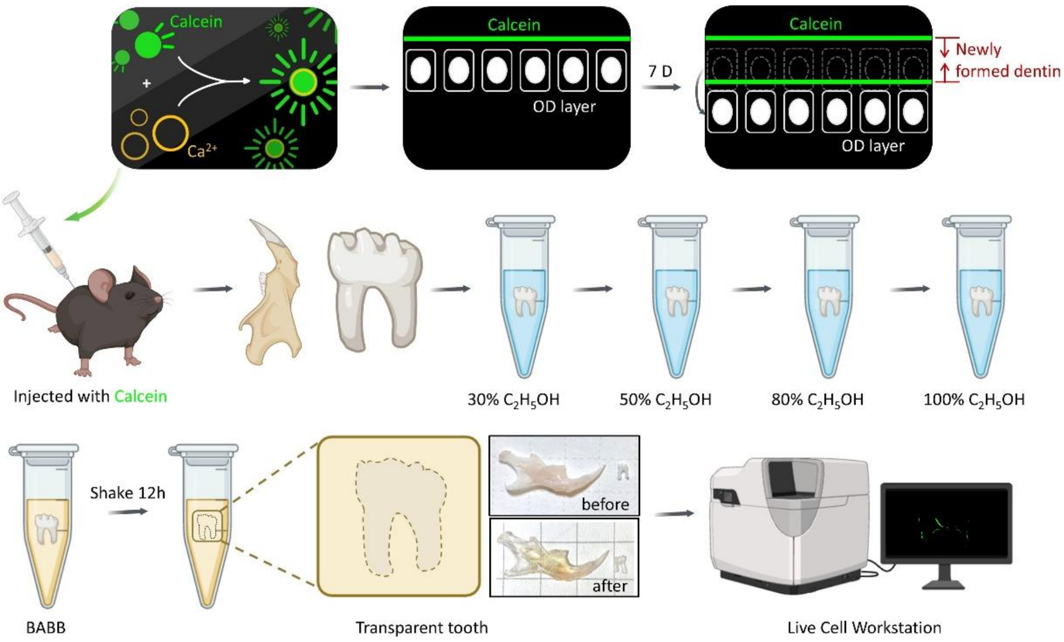

BABB, the transparent working solution was prepared by mixing benzyl benzoate (Sigma-Aldrich) and benzyl alcohol (Sigma-Aldrich) in a 2:1 volume ratio, referring to Fig. 5D.

SUPPLEMENTAL TABLES

Table S1. Characteristics of enrolled cases by group.

	Young	Aged
Population (Female/Male)	55/29	25/14
Age (Min-Max, Mean±SEM)	18-40, 28.12±0.63	60-87, 70.79±1.27
Arch (Maxillary/Mandibular)	66/57	51/58
Position (Anterior/Posterior)	10/113	28/81
Outcome (IP/Pulp survival)	12/111	34/75

Table S2. Summary of the association between age and irreversible pulpitis progression.

Model	Covariate adjustment	Age effect estimate (OR, 95%CI)	*P* value
Crude	None	1.0215 (1.0060-1.0382)	0.0077
Multivariable	Sex, dental arch, and tooth position	1.0180 (1.0020-1.0360)	0.0330
Mixed-effects	Sex, dental arch, and tooth position; patient-level random intercept included	1.0403 (1.0383-1.0422)	< 0.001
Sensitivity	One tooth per patient retained, iterating 1000 times	Mean OR = 1.0294*	Median $p = 0.0135$

* OR > 1 in 100% of iterations and statistically significant in 95.3% of iterations.

Table S3. 108 core DnDEGs in human dental pulp MSC senescence.

Genes	Uniprot Annotation	Genes	Uniprot Annotation
MCM10	Nucleus	TROAP	cytosol
ORC1	Nucleus	MCM5	Cytosol
LMNB1	Nucleus	DLGAP5	Cytoskeleton
CDC45	Nucleus	RRM2	Cytosol
E2F8	Nucleus	PBK	Nucleus
CENPA	Nucleus	KNL1	Nucleus
MKI67	Nucleus	BUB1	Nucleus
SKA1	Cytoskeleton	HJURP	Nucleus
EXO1	Nucleus	ERCC6L	Cytosol
CDT1	Nucleus	CEP55	Cytoskeleton
KIF18B	Cytoskeleton	CDCA5	cytosol
NCAPH	Cytoskeleton	CDCA8	Cytoskeleton
PLK4	Cytoskeleton	EZH2	Nucleus
GINS1	Nucleus	TPX2	Cytoskeleton
AURKB	Cytoskeleton	KIF15	Cytoskeleton
SPC24	Nucleus	KIF2C	Nucleus
SKA3	Cytoskeleton	ESPL1	Cytoskeleton
NCAPG	Nucleus	SPAG5	Cytoskeleton
NUF2	Nucleus	NEK2	Cytoskeleton
MCM2	Nucleus	CENPM	Cytoskeleton
PLK1	Cytoskeleton	TTK	Cytoskeleton
SPC25	Nucleus	RAD54L	Nucleus
GTSE1	Cytoskeleton	ZNF367	Nucleus
BUB1B	Cytoskeleton	TACC3	Cytoskeleton
KIF11	Cytoskeleton	DEPDC1	Nucleus
ASPM	Cytoskeleton	CENPW	Nucleus
WDR76	Nucleus	CENPF	Cytoskeleton
UBE2C	Cytosol	NEIL3	Nucleus
CDC20	Cytoskeleton	CCNA2	Nucleus
UHRF1	Nucleus	SHCBP1	Cytoskeleton
CCNF	Cytoskeleton	DEPDC1B	Cytosol
NDC80	Nucleus	KIF4A	Cytoskeleton
TCF19	Nucleus	NUSAP1	Cytoskeleton
RAD51AP1	Nucleus	MAD2L1	Cytoskeleton
ZWINT	Nucleus	UBE2T	Nucleus
TRIP13	Nucleus	CDKN2C	Cytoskeleton
MND1	Nucleus	CHTF18	Nucleus
RACGAP1	Plasma membrane	APOBEC3B	Nucleus
FAM83D	Cytoskeleton	CKS1B	Nucleus
SOX11	Nucleus	CDKN3	Cytosol

TYMS	Nucleus	KIF20A	Golgi apparatus
PRC1	Cytoskeleton	SMC4	Nucleus
SGO2	Nucleus	CENPH	Nucleus
HMMR	Plasma membrane	MELK	Plasma membrane
CCNB2	Cytoskeleton	GPSM2	Plasma membrane
TOP2A	Nucleus	TK1	Cytosol
ANLN	Cytoskeleton	GAS2L3	Cytoskeleton
CDCA3	Cytosol	AURKA	Cytoskeleton
KIF14	Cytoskeleton	PRR11	Nucleus
NCAPG2	Nucleus	POC1A	Cytoskeleton
CCNB1	Nucleus	KIF23	Cytoskeleton
OIP5	Cytoskeleton	BRIP1	Nucleus
BIRC5	Cytoskeleton	PSRC1	Cytoskeleton
TMPO	Nucleus	BARD1	Nucleus

Table S4. RT-qPCR primers.

Gene		Sequence (5'-3')
GAPDH	Forward	GCTCTCTGCTCCTCCTGTTCG
	Reverse	GCGAACACATCCGGCCTGC
NFATC1	Forward	GCATCACAGGGAAGACCGTGTC
	Reverse	GAAGTTCAATGTCGGAGTTTCTGAG
ALPL	Forward	GACCTCCTCGGAAGACACTC
	Reverse	TGAAGGGCTTCTTGTCTGTG
COL1A1	Forward	TCTAGACATGTTCAGCTTTGTGGAC
	Reverse	TCTGTACGCAGGTGATTGGTG
SP7	Forward	TCTCCATCTGCCTGACTCCT
	Reverse	AGCGTATGGCTTCTTTGTGC
IBSP	Forward	CAGGCCACGATATTATCTTTACA
	Reverse	CTCCTCTTCTTCCTCCTCCTC
SPP1	Forward	ATGATGGCCGAGGTGATAGT
	Reverse	ACCATTCAACTCCTCGCTTT
TNFRSF11B	Forward	GTGTGCGAATGCAAGGAAGG
	Reverse	CCACTCCAAATCCAGGAGGG
DSPP	Forward	CAACCATAGAGAAAGCAAACGCG
	Reverse	TTTCTGTTGCCACTGCTGGGAC
